# Structural modeling and functional characterization of a novel gain-of-function TLR8 variant causing severe inflammatory syndrome

**DOI:** 10.1172/jci.insight.187422

**Published:** 2026-02-23

**Authors:** Nikolaos T. Skenteris, Elisa Luttermann, Sanjana Nair, Ioannis Evangelakos, Maria Pujantell, Marie Eggers, Fabian Hausmann, Marleen Bérouti, Benedetta Padoan, Felix J. Flomm, Janna M. Claussen, Benjamin Grünhagel, Anika Salfelder, Brigitte Beifuss, Saskia Biskup, Patrick Blümke, Katrin Rading, Heike Hildebrandt, Urte Matschl, Silke Giesemann-Jansen, Jana Hennesen, Viacheslav O. Nikolaev, Michael Kutsche, Christian Kubisch, Friedrich Koch-Nolte, Nicola M. Tomas, Eva Tolosa, Marc Lütgehetmann, Felix R. Stahl, Veit Hornung, Madeleine J. Bunders, Christian Schlein, Maya Topf, Ina Kötter, Marcus Altfeld

**Affiliations:** 1 Research Department of Virus Immunology, Leibniz Institute of Virology, Hamburg, Germany.; 2 Clinic for Rheumatology and Immunology, Auenlandklinik Bad Bramstedt, Bad Bramstedt, Germany.; 3 Research Department of Integrative Virology, Leibniz Institute of Virology, Hamburg, Germany.; 4 Centre for Structural Systems Biology, Hamburg, Germany.; 5 Institute of Human Genetics,; 6 Institute of Immunology,; 7 III. Department of Medicine, and; 8 Hamburg Center for Kidney Health, University Medical Center Hamburg-Eppendorf, Hamburg, Germany.; 9 Gene Center and Department of Biochemistry, Ludwig Maximilian University of Munich, Munich, Germany.; 10 Zentrum für Humangenetik, Tübingen, Germany.; 11 Center for Genomics and Transcriptomics, Tübingen, Germany.; 12 Technology Platform Next Generation Sequencing, Leibniz Institute of Virology, Hamburg, Germany.; 13 Institute of Experimental Cardiovascular Research;; 14 Hamburg Center for Translational Immunology;; 15 German Center for Child and Adolescent Health (DZKJ), partner site Hamburg; and; 16 Institute of Medical Microbiology, Virology and Hygiene, University Medical Center Hamburg-Eppendorf, Hamburg, Germany.; 17 German Center for Child and Adolescent Health (DZKJ), partner site Munich, Munich, Germany.; 18 Institute for Molecular Virology and Tumorvirology, University Medical Center Hamburg-Eppendorf, Hamburg, Germany.; 19 German Center for Child and Adolescent Health (DZKJ), partner site Hamburg; Leibniz Institute of Virology, Hamburg, Germany.

**Keywords:** Genetics, Immunology, Infectious disease, Genetic variation, Molecular genetics, NF-kappaB

## Abstract

With the increasing use of genetic sequencing to investigate inborn errors of immunity, rare variants are frequently identified, yet their clinical relevance often remains uncertain. Establishing pathogenicity requires a multidisciplinary approach that integrates genetic, structural, functional, and clinical data. Here, we used such a strategy to investigate a previously unreported hemizygous missense variant — alanine (A) to threonine (T) at residue 518 — in Toll-like receptor 8 (TLR8), identified in 2 male siblings with recurrent infections and systemic inflammation, characterized by a proinflammatory immune signature and B cell dysregulation. Functional studies showed that the TLR8 A518T variant enhanced NF-κB activation and increased secretion of proinflammatory cytokines compared with WT TLR8 upon stimulation, consistent with a gain-of-function effect. Protein degradation and turnover assays revealed reduced abundance of the mutant TLR8 protein due to faster turnover and increased proteasomal degradation. Computational modeling predicted enhanced structural stabilization of the active TLR8 homodimer interface via additional water-mediated hydrogen bonds introduced by the A518T substitution. Together, these findings integrating structural modeling with functional assays identify a novel TLR8 ligand-specific gain-of-function mutation resulting in complex immunopathology in 2 siblings.

## Abstract 2

A newly discovered TLR8 gene mutation causes immune overactivation and inflammation in two siblings, linking genetic change to immune system dysfunction and disease.

## Graphical abstract

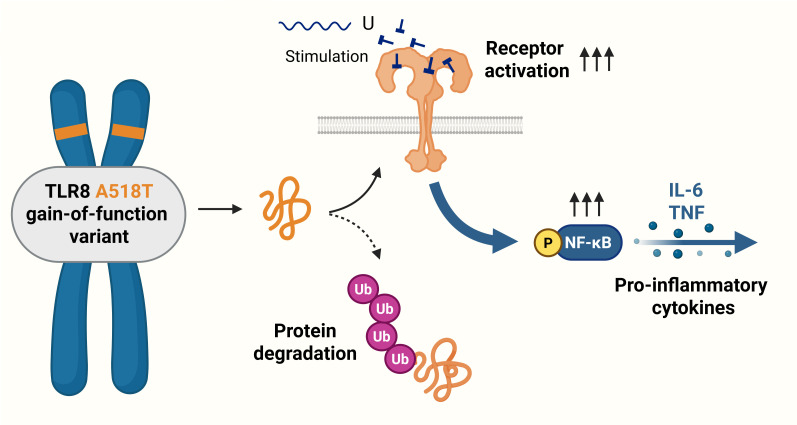

## Introduction

Precise identification of pathogenic genetic variants underlying inborn errors of immunity (IEI), which are manifested by symptoms including recurrent infections, autoimmunity, and chronic inflammation, is essential for the discovery and diagnosis of rare diseases as well as targeted treatment of carriers (1). With the prevalent use of genetic sequencing to diagnose individuals with rare genetic diseases, such as IEI, an increasing number of previously unidentified genetic variants are being discovered, whereas the functional consequences are unknown. In recent years, over 485 variants known to cause IEI have been officially identified (1, 2); however, many patients still experience substantial delays in reaching a definitive diagnosis after initial clinical presentation. Improved precise functional predictions for genetic variants will benefit diagnostic accuracy and treatment.

Although experimental analyses to understand the function of individual genetic variants have been the cornerstone of research and clinical care of individuals with IEI, the strength of computational modeling predicting the structure and function of specific proteins and variants is increasingly recognized (3). Advanced accurate computational methodologies hold promise in narrowing the translational disparity between genetic variants and functional consequences by analyzing patterns within biological data in order to predict the pathogenicity of unannotated variants (3). In current models, pathogenicity is evaluated based on the variation between reference and alternate sequences. Computational modeling predicts the structural and functional impact of genetic variants based on deep learning algorithms (4). Here, we applied these techniques along with functional assays on a previously unreported putatively pathogenic variant in the *TLR8* gene observed in 2 related male individuals.

Recent genetic studies have revealed a broad spectrum of Toll-like receptor 8 (TLR8) variants in human populations (5–8), several of them linked to IEI. TLR8 is a pathogen-associated molecular pattern–recognizing (PAMP-recognizing) receptor that detects viral and bacterial RNA, orchestrating immune activation pathways essential for pathogen clearance and immune surveillance (9–12). In addition to external PAMPs, TLR8 can respond to endogenous stimuli such as microRNAs (13) or autoantigens (14), and drive autoimmunity (15). Binding of ligands to preformed TLR8 homodimers induces conformational changes, bringing the C-terminal domains closer together (16). This event subsequently triggers downstream signaling pathways and leads to the production of a variety of nuclear factor-κB–mediated (NF-κB–mediated) proinflammatory cytokines, interferon regulatory factor–induced (IRF-induced) interferons (IFNs), and other effector molecules essential for antiviral and antibacterial defense (17–19).

In this study, we present a comprehensive investigation of a previously unreported missense variant in the *TLR8* gene (c.1552G>A, p.A518T), identified in 2 male siblings with recurrent infections and inflammatory symptoms. To characterize the pathogenic potential of this variant, we used an integrative approach combining clinical evaluation, gene expression profiling, computational structural modeling, and in vitro functional assays. Plasma and immune profiling revealed a proinflammatory immune signature and marked B cell dysregulation in the affected individuals. Functional assays demonstrated increased TLR8-mediated NF-κB phosphorylation and elevated secretion of proinflammatory cytokines following stimulation, consistent with gain of function. In contrast, protein turnover studies showed reduced baseline abundance of the A518T variant due to faster degradation and increased proteasomal susceptibility. Structural modeling predicted that the A518T substitution enhances stabilization of the active TLR8 homodimer interface through the formation of additional water-mediated hydrogen bonds. Collectively, these findings demonstrate two consequences of the TLR8 A518T variant: ligand-dependent gain of function driving inflammation, alongside reduced overall TLR8 protein abundance as a consequence of accelerated protein turnover and degradation, ultimately resulting in the complex immunopathology observed in the two siblings.

## Results

### Clinical phenotype of study participants.

**Figure 1:**
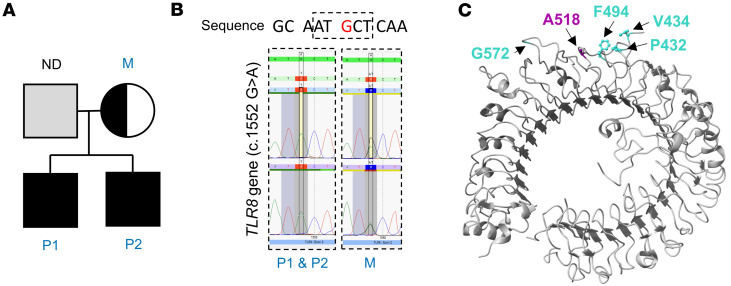
Investigation of the germline TLR8 c.1552G>A (p.A518T) variant. (**A**) The family tree depicts individuals with the TLR8 variant. Solid black boxes represent males P1 and P2 with the germline variant, while half-black circles indicate heterozygous female carriers. ND, not determined. (**B**) Sanger sequencing of the family members’ DNA confirms the presence of the TLR8 variant. (**C**) Leucine-rich repeat (LRR) TLR8 with the Z-loop (PDB ID: 3W3G). The A518 variant in LRR 16 is highlighted in purple. Previously reported variants from Aluri et al. (5), Fejtkova et al. (6), and Liang et al. (8) are denoted in cyan.

In this study, 2 male siblings were included, denoted as participant 1 (P1, age 27 years) and participant 2 (P2, age 24 years); as well as their mother (M, age 48 years) (Figure 1A). Their heights and weights were normal at birth. Both siblings exhibited severe inflammatory symptoms in the first year of life and were diagnosed with severe anemia requiring red blood cell transfusions. Initially, autoimmune vasculitis was suspected based on recurrent cerebral infarctions and peripheral hypoperfusion. Both siblings displayed an increased susceptibility to infections, with recurring respiratory infections requiring antibiotic treatment. In addition, frequent episodes of high fever were observed without clear signs of infections, suggesting an inflammatory syndrome. Inflammatory markers, such as CRP, were increased in both P1 and P2 (Supplemental Figure 1A; supplemental material available online with this article; https://doi.org/10.1172/jci.insight.187422DS1). Laboratory analyses revealed anemia, thrombocytopenia, and a severe leukopenia (<100 cells per μL). Although small changes in the bone marrow smears were observed in both individuals, erythropoiesis was not decreased, nor thrombopoiesis at early time points. Immunoglobulin deficiencies were observed in P1 (IgA, IgG, and IgM) and in P2 (IgM and IgG) (Supplemental Figure 1B). Furthermore, both siblings presented lymphadenopathy and splenomegaly, and exhibited delayed psychoneuromotor development and intellectual disability. Moreover, spastic hemiparesis resulting from cerebral ischemia limited their mobility.

Following their initial diagnosis of cerebral vasculitis and polyarteritis nodosa, both individuals underwent immunosuppressive therapies. P1 received treatment with corticosteroids and additional immunosuppressive drugs, including cyclophosphamide, azathioprine, and mycophenolic acid (Supplemental Figure 1C). The therapies had to be discontinued because of either lack of therapeutic response or severe toxicity at the age of 13. From the age of 15 years, P1 received infliximab, resulting in improvement of clinical symptoms lasting up to the present. P2 has been receiving corticosteroids since birth to control inflammatory syndrome and was treated with infliximab since the age of 17 (Supplemental Figure 1C). The latter had to be subsequently switched to adalimumab because of an allergic reaction to infliximab. Furthermore, intravenous immunoglobulin (IVIg) replacement therapy was administered to both siblings due to low serum immunoglobulin levels and recurrent infections. While P1 demonstrated an adequate increase in immunoglobulin levels, allowing for cessation of therapy after 8 years, P2 continues to exhibit low levels of IgM and IgG and receives repeated IVIg injections (Supplemental Figure 1B). In summary, P1 and P2 presented in early childhood with an inflammatory syndrome and immune deficiency, with P2 presenting with a more severe clinical phenotype. The clinical symptoms improved after initiation of immunosuppressive therapy.

### Identification of the germline TLR8 c.1552G>A (p.A518T) variant in both siblings and their mother.

To identify the underlying cause of the chronic inflammatory syndrome in these two male siblings, targeted exome sequencing was performed and identified a shared hemizygous missense variant (cytogenetic: Xp22.2; chromosome X, position 12920592 [GRCh38.p14], c.1552G>A) in the X chromosome–encoded *TLR8* gene (NM_138636.5), for which the mother was heterozygous (Figure 1B). The variant is now registered in the NCBI’s Single Nucleotide Polymorphism Database (dbSNP) and ClinVar with the identifiers rs2147259148 and RCV002263534.1, respectively. The c.1552G>A variant leads to a substitution of threonine (T) for alanine (A) at position 518 in the leucine-rich repeat of the TLR8 protein (NP_619542.1; p.A518T) (Figure 1C). The variant was not present in the Genome Aggregation Database (gnomAD) (20), and it was predicted to be proxy-deleterious by Combined Annotation Dependent Depletion (CADD; GRCh38-v1.4) score (Supplemental Table 1) (21). To complement the analysis, we also applied additional in silico prediction tools. PolyPhen-2 classified the variant as benign in both HumDiv (score: 0.043; sensitivity: 0.94; specificity: 0.83) and HumVar (score: 0.004; sensitivity: 0.98; specificity: 0.35) models (22). Similarly, MutationTaster predicted the variant to be benign (22), and Sorting Intolerant from Tolerant (SIFT) classified it as tolerated (22). While all 3 tools indicated that the variant is unlikely to be deleterious, these prediction tools do not assess potential gain-of-function effects or provide insights into structural or functional changes in protein activity. Further genetic analysis of the subjects revealed a second, not previously reported variant only in P2 in the DnaJ heat shock protein family (Hsp40) member C21 (*DNAJC21*) gene (chromosome 5, position 34929752–34929753 AC/A [GRCh37.p13], NM_001012339.3:c.-171del, rs1266840568, RCV002263372.13, heterozygous). Given the shared mutation with the TLR8 protein between the two related male individuals, we subsequently investigated the immune implications of the TLR8 A518T variant.

### Systemic immune activation in individuals with the TLR8 A518T variant.

**Figure 2:**
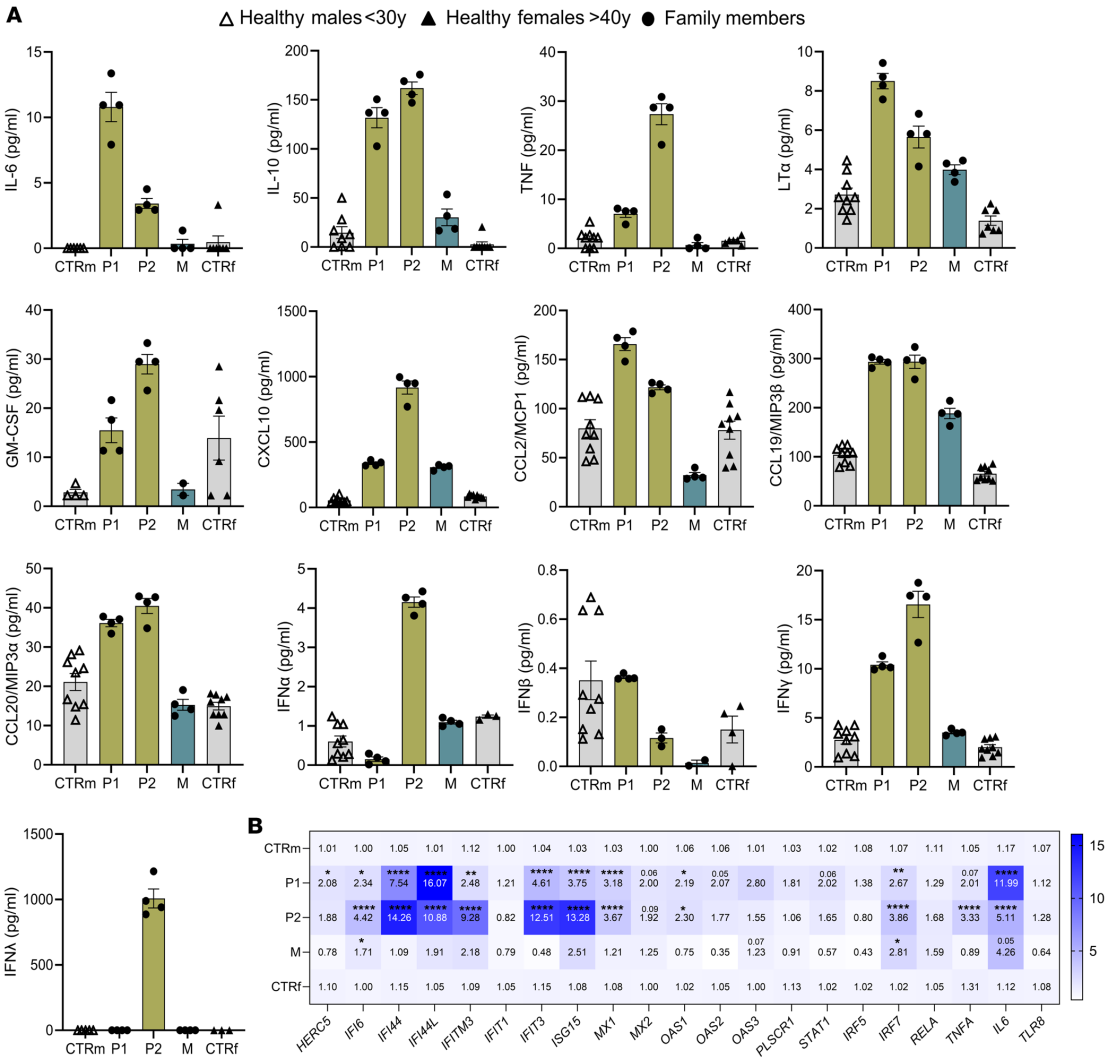
Systemic immune activation in siblings with the TLR8 A518T variant. (**A**) Plasma analysis of several proinflammatory cytokines, growth factors, and chemokines in family members (P1, P2, and M; performed in quadruplet) in comparison with healthy male individuals less than 30 years old (*n* = 3–4; technical replicates) and healthy female individuals greater than 40 years old (*n* = 3–4; technical replicates). Data are expressed as mean with SEM. (**B**) Gene expression analysis in family members (P1, P2, and M) in comparison with healthy male individuals less than 30 years old (*n* = 3) and healthy female individuals greater than 40 years old (*n* = 3) of IFN-stimulating genes (ISGs), cytokines, and transcription factors. The number in the center of each square represents the mean value of relative quantification, while the values above or the asterisks indicate statistical significance. Statistical significance between groups was assessed using a 2-way ANOVA multiple-comparison test. Data represent mean ± SEM. Differences between groups were considered significant at *P* values less than 0.05 (*P < 0.05, **P ≤ 0.01, ****P ≤ 0.0001).

To further characterize the inflammatory syndrome of the two siblings with the TLR8 A518T variant, plasma profiling was performed at the age of 25 and 22 for P1 and P2, respectively. Significantly elevated levels of several proinflammatory cytokines, including IL-6 and TNF as well as growth factors like GM-CSF and chemokines such as CXCL10, CCL2, CCL19, and CCL20, were detected (Figure 2A and Supplemental Figure 2). Notably, P2 also exhibited increased serum levels of IFN-α (type I IFN) and IFN-λ (type III IFN), while both individuals showed normal levels of IFN-β. Both individuals additionally displayed elevated levels of IFN-γ, the sole member of the type II IFN family. This corresponded to an upregulation in the expression levels of several IFN-stimulated genes (ISGs), as quantified by quantitative PCR in peripheral blood mononuclear cells (PBMCs) from both individuals compared with age-matched controls (Figure 2B). Additionally, elevated mRNA levels of the transcription factor *IRF7* were observed in both cases. Notably, the *TLR8* mRNA expression levels were comparable between the siblings and the healthy controls. Collectively, these findings suggest systemic inflammation in the siblings with the TLR8 A518T variant. Despite the increased expression of some ISGs in the mother’s cells, X chromosome inactivation analysis performed on the mother’s PBMCs using quantitative PCR for 2 independent X-linked genes, androgen receptor (*AR*) and retinitis pigmentosa 2 (*RP2*), revealed a random inactivation pattern, with ratios of 0.53 and 0.49, respectively, making significant skewing or escape from inactivation unlikely (23).

### Altered immune cell composition and innate immune dysregulation in individuals with the TLR8 A518T variant.

The clinical immunological profile of individuals carrying the TLR8 A518T variant revealed altered cellular composition and variability among the two affected siblings (P1 and P2) and to a more limited extent in their mother, as summarized in Table 1, Table 2, and Table 3. Both siblings had relatively low to severely decreased numbers of T cells, with CD4^+^ T cells affected in both participants and CD8^+^ T cells also affected in P2, resulting in an inverse CD4/CD8 ratio (<2). Additionally, P2 and M had a slight reduction in the frequency of T regulatory cells in the peripheral blood. A dysregulated B cell phenotype was observed in both P1 and P2, with diminished numbers of memory B cells (CD27^+^CD21^+^ resting, CD27^+^CD21^–^ activated, and CD27^–^CD21^–^ tissue-like memory B cells) and CD27^+^CD38^+^ plasmablasts. Both siblings also showed a reduction in activated CD21^lo^CD38^–^ B cells.

Extended immunophenotyping by multiparametric flow cytometry confirmed dysregulation of several immune populations (Supplemental Figures 3–5). P1 and P2 exhibited slightly increased frequencies of CD14^+^CD16^+^ intermediate monocytes, while a decreased frequency of non-classical monocytes was observed in P2 (Supplemental Figure 4). A reduction in the frequency of CD141^+^ dendritic cells and CD123^+^HLA-DR^+^ plasmacytoid dendritic cells was observed in both siblings (Supplemental Figure 5), compared with age-matched controls. These analyses demonstrate dysregulation of innate immune cell populations in the individuals carrying the TLR8 A518T variant.

### Impaired T and B cell function leading to defective humoral immunity.

**Figure 3:**
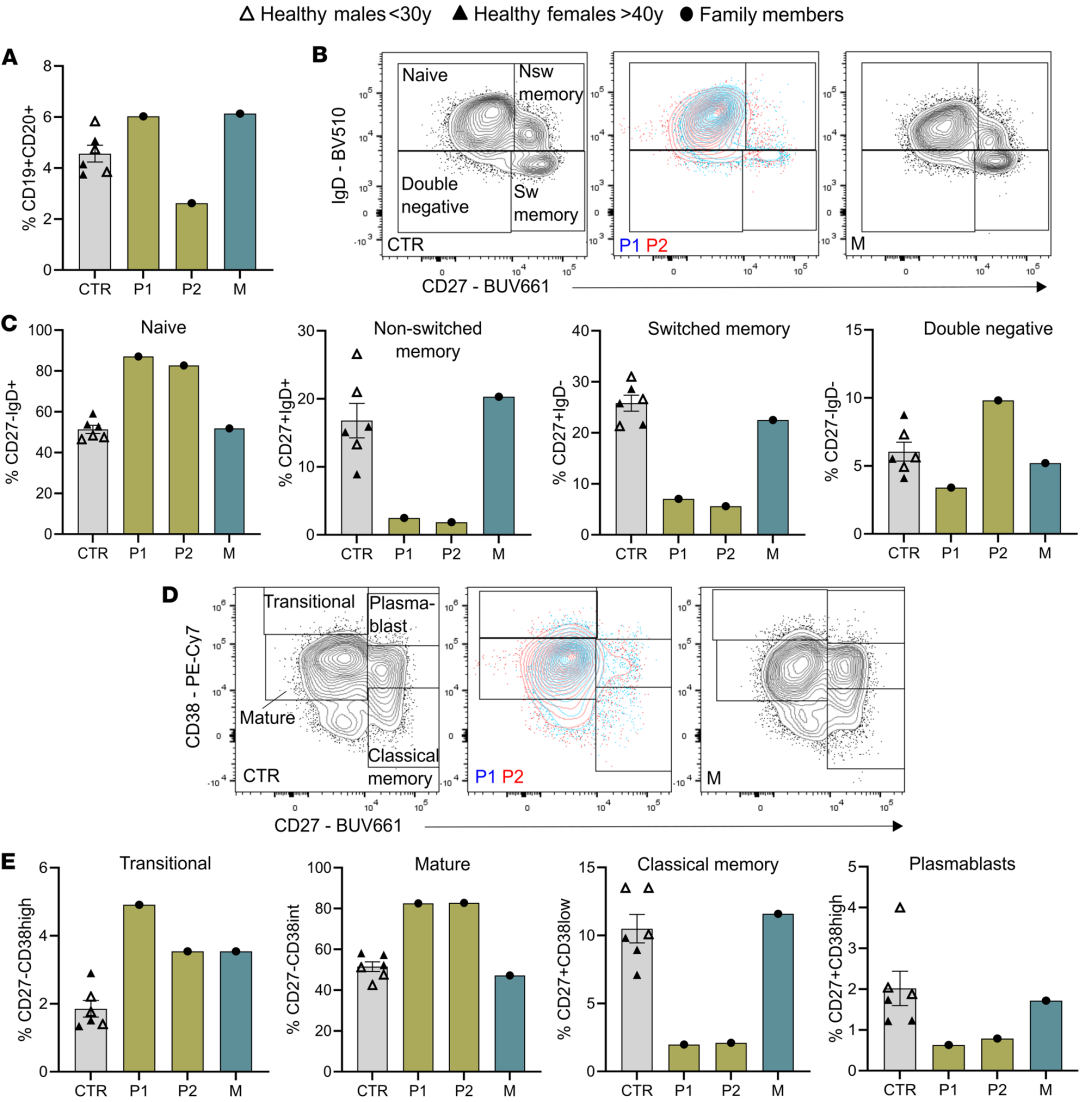
B cell dysregulation in siblings with TLR8 A518T variant. (**A**) Percentage of CD19^+^CD20^+^ B cells in family members relative to healthy male (*n* = 3) and female (*n* = 3) individuals (CTR). (**B**–**E**) Representative flow cytometry plots, along with the relative frequencies, of B cell subtypes in family members relative to healthy male (*n* = 3) and female (*n* = 3) individuals (CTR). Data represent mean ± SEM.

Previous studies suggested that chronic TLR activation and elevated proinflammatory cytokines can impair B cell development and circulation (24, 25). Flow cytometry analyses revealed that P2 had a lower frequency of CD19^+^CD20^+^ B cells, consistent with systemic inflammation (Figure 3A). Both P1 and P2 showed increased frequencies of naive (CD27^–^IgD^+^), mature (CD27^–^CD38^int^), and transitional (CD27^–^CD38^hi^) B cells, alongside reduced frequencies of memory B cells (classical CD27^+^CD38^lo^, switched CD27^+^IgD^–^, and non-switched CD27^+^IgD^+^) and antibody-secreting CD27^+^CD38^hi^ plasmablasts (Figure 3, B–E), indicating impaired B cell maturation and differentiation. A much milder B cell dysregulation was observed in their asymptomatic mother.

**Figure 4:**
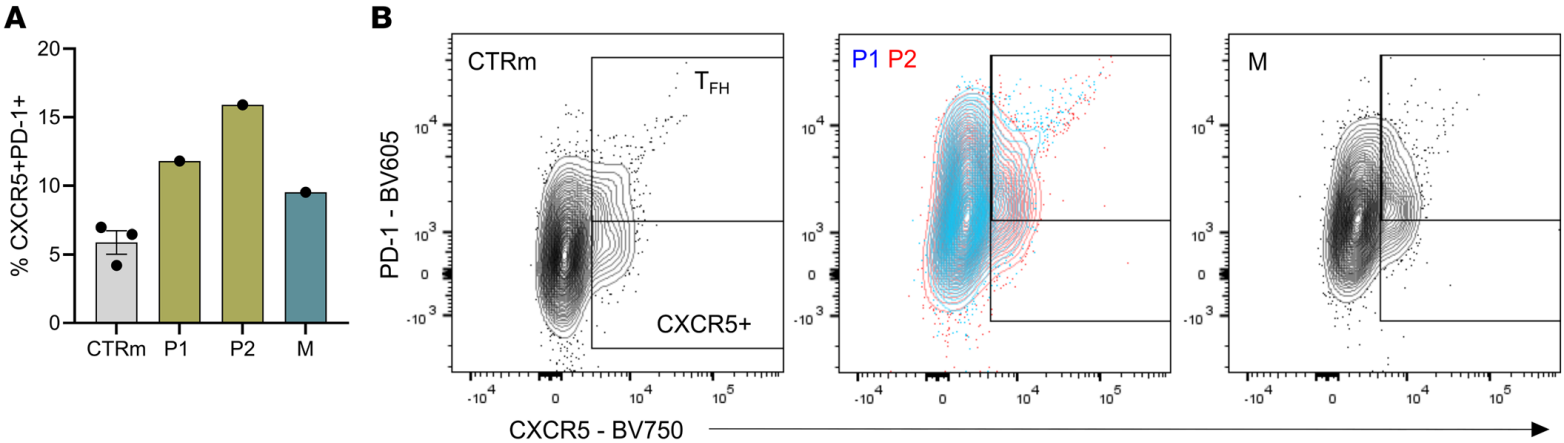
Expansion of T follicular helper cells in siblings with the TLR8 A518T variant. Frequencies of T follicular helper cells (Tfh: CXCR5^+^PD-1^+^), along with their representative flow cytometry plots, in family members (P1, P2, and M) relative to healthy male (*n* = 3) individuals (CTRm). Data represent mean ± SEM for healthy male controls.

T cell subset analyses revealed T cell exhaustion features, including elevated PD-1 expression and inverted CD4/CD8 ratios (Supplemental Figure 6, A and B). Upon CD3/CD28 stimulation, CD4^+^ T cells from variant carriers showed reduced viability compared with age-matched controls (Supplemental Figure 6C). While expression levels of ICOS and CD40L on CD4^+^ T cells and CXCR5^+^PD-1^+^ T follicular helper (Tfh) cells were comparable between variant carriers and healthy controls (Supplemental Figure 6, D and E), further immunophenotyping of the CD4^+^ and CD8^+^ cell subsets revealed phenotypic heterogeneity among the siblings and their mother, with the latter displaying a profile more similar to that of P2 (Supplemental Figure 6, F and G). Notably, the frequency of CXCR5^+^PD-1^+^ Tfh cells was elevated in both P1 and P2, based on analyses of enriched CD4^+^ T cells (Figure 4 and Supplemental Figure 7), suggesting a skewed T helper cell profile in conjunction with impaired B cell maturation.

**Figure 5:**
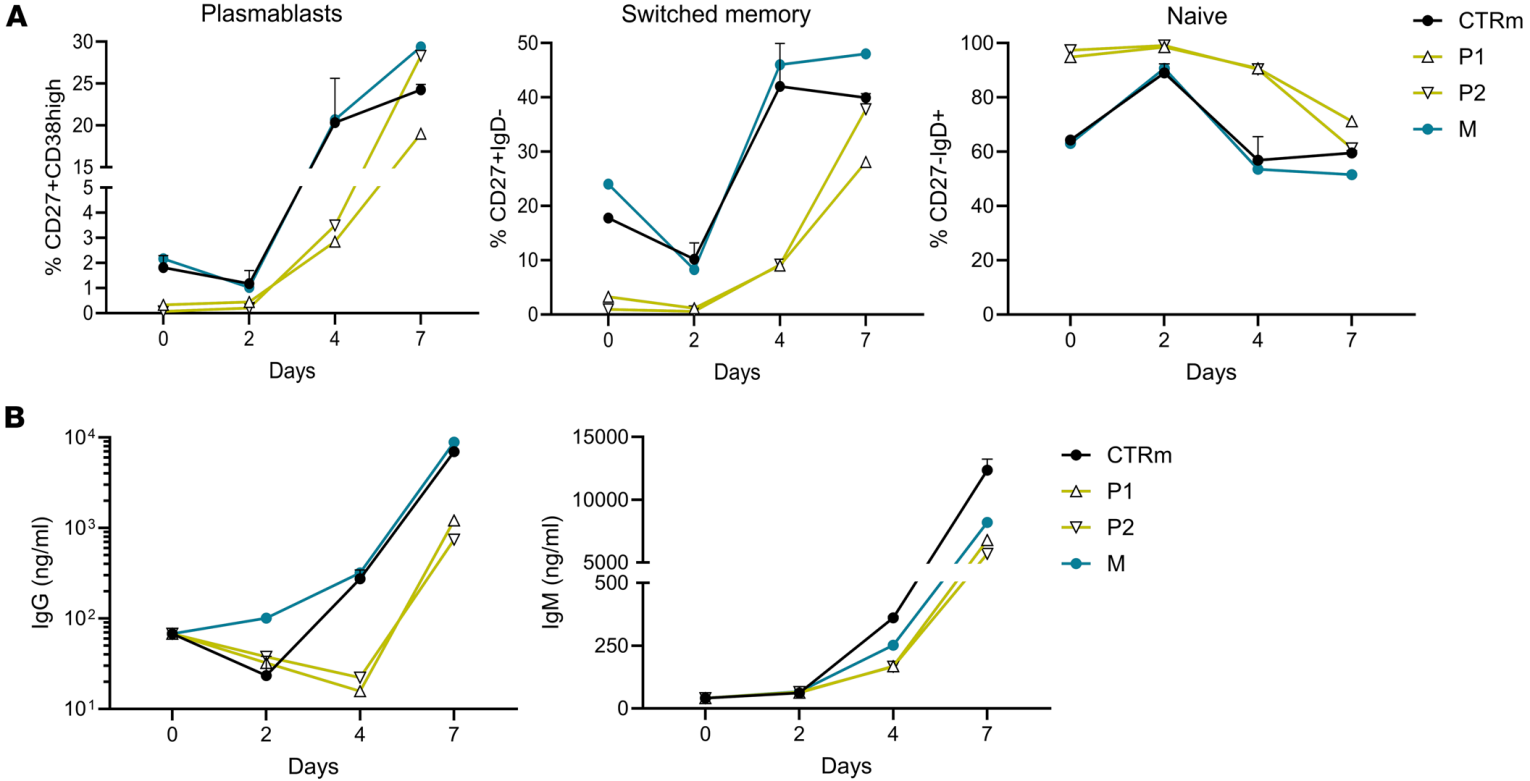
Impaired B cell differentiation, memory and plasmablast formation, and immunoglobulin production in siblings with the TLR8 A518T variant. (**A**) Quantification of plasmablasts, switched memory, and naive B cells from CD40L-stimulated B cells over time in family members (P1, P2, and M) relative to healthy male (*n* = 3) individuals (CTRm). (**B**) Quantification of the secreted IgG and IgM levels in the supernatants of CD40L-stimulated B cells over time in family members (P1, P2, and M) relative to healthy male (*n* = 3) individuals (CTRm). Data represent mean ± SEM for healthy male controls.

Functional assays showed delayed in vitro differentiation of B cells from P1 and P2 into plasmablasts and memory cells following CD40L stimulation, with persistently high levels of naive B cells (Figure 5A and Supplemental Figures 8 and 9) and reduced IgG/IgM production (Figure 5B). Consistent with these findings, P1 — who discontinued IVIg therapy — demonstrated poor antibody responses to several vaccines (Table 2). Altogether, these findings demonstrate that the 2 siblings with the TLR8 A518T variant exhibit a dysregulated immune profile marked by T cell exhaustion, increased frequencies of Tfh cells, and defective B cell maturation and differentiation. These immune alterations result in reduced memory B cells, plasmablasts, and antibody production, and were associated with impaired humoral immunity.

### Computational modeling reveals that the A518T variant renders the active state of the TLR8 homodimer structurally more stable.

**Figure 6:**
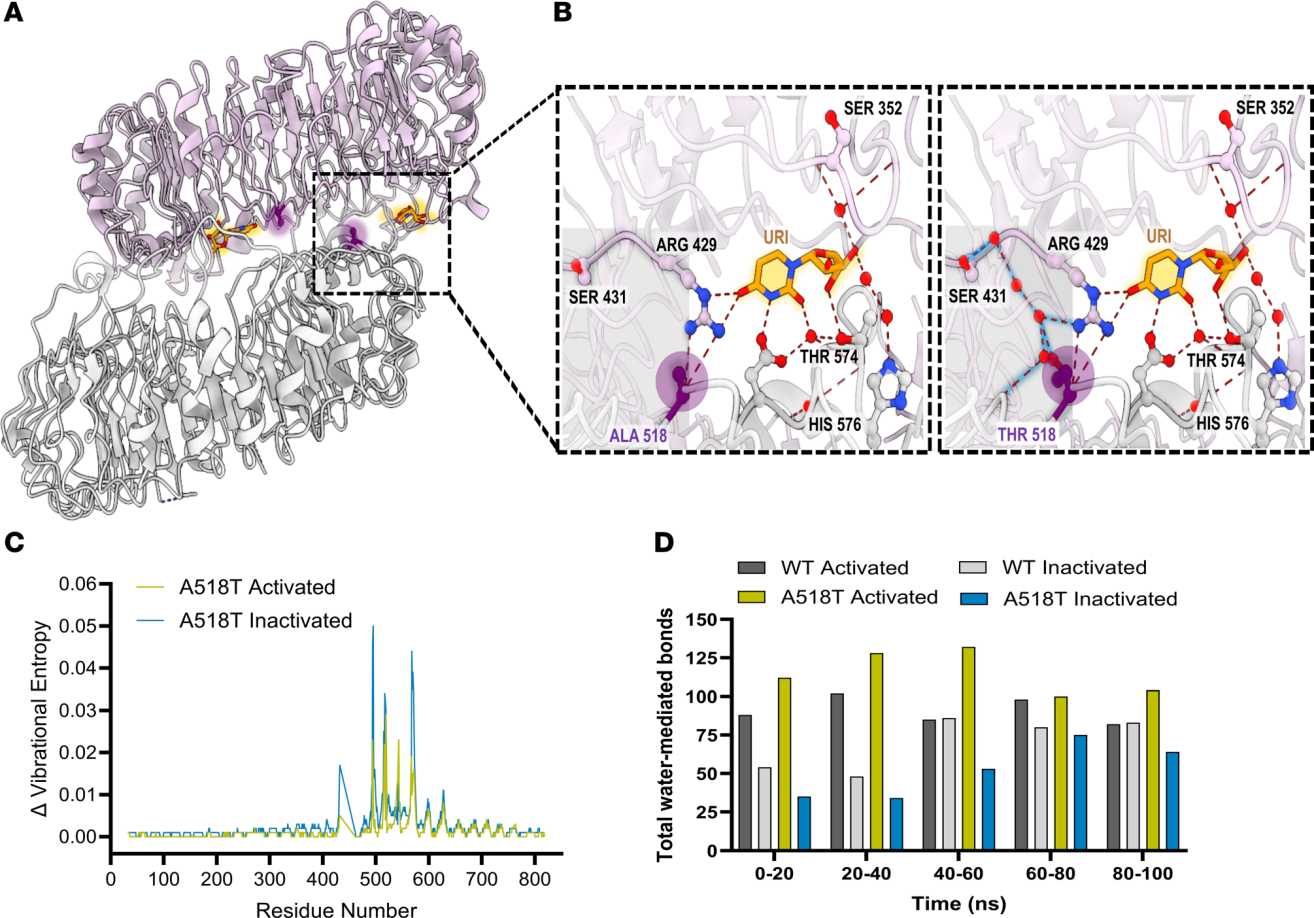
Computational modeling indicates that the TLR8 A518T variant stabilizes the structure of the active conformation of the receptor homodimer. (**A**) Ribbon diagram of the side view of TLR8 receptor homodimer (PDB ID: 4R08) with the 2 chains colored gray and pink, and residue 518 indicated in a purple halo, positioned at the interface of the homodimer and the uridine residue in ball-and-stick representation in orange with an orange halo. (**B**) Comparison of hydrogen bonds in the WT protein (left) and A518T variant (right). The hydrogen bonds that are conserved in both proteins are shown as red dashed lines, and the additional hydrogen bonds formed by the A518T variant are shown as black dashed lines and a blue halo. A gray rectangle highlights the differences in hydrogen bonding patterns in the WT and A518T variant proteins. The uridine residue (URI) is represented with ball and stick in orange with an orange halo around it, and the atoms are colored according to the elements with red and blue spheres representing oxygen and nitrogen, respectively. The hydrogen-bonded residues are numbered and shown with ball-and-stick representation with their respective 3-letter codes. (**C**) Comparison of the Δ vibrational entropy energy of A518T variant with respect to the WT protein in the ligand-bound activated state (green) and the unbound inactivated state (blue) with the residue numbers on the *x* axis, calculated by DynaMut. (**D**) Comparison of water-mediated interchain hydrogen bonds in the WT protein in the activated state (dark gray), WT protein in the inactivated state (light gray), A518T variant in the activated state (blue), and A518T variant in the inactivated state (green), based on molecular dynamics simulations with OpenMM.

The clinical data illustrated that the TLR8 p.A518T mutation potentially reflected a gain-of-function mutation in TLR8, linked to significant immune dysregulation. To determine whether structural modeling of the TLR8 receptor, using the genetic information, would provide reliable predictions of the functional consequences, we performed structural modeling using MODELLER v10.3 (26). Modeling prediction of proteins encoded by mutated genes on the X chromosome tends to be more accurate in males, particularly for proteins forming homodimers. This is because males have only one X chromosome, resulting in homodimers formed exclusively from 2 copies of the mutated protein, which simplifies the functional prediction. Based on structural studies of TLR8, it has been observed that upon ligand (uridine) binding, conformational changes occur in preformed TLR8 dimers, bringing the C-terminal domains closer together, thus initiating downstream signaling (11, 16). The 3-dimensional structure of the TLR8 protein showed that residue 518 is located on the protein-protein interface, and in close proximity to the uridine-binding site of the homodimer (Figure 6A). The predicted structural model of the variant revealed that substitution of the alanine (A) with threonine (T) formed additional water-mediated hydrogen bonds at the protein-protein interface in the presence of uridine agonist, thereby stabilizing the structure of the active homodimer (Figure 6B).

This finding was supported by the normal mode analysis of the active and the inactive conformations of the TLR8 mutant in regard to the respective WT states, using DynaMut (27). The free energy change (ΔΔG) between the WT TLR8 protein and the variant protein in the inactive conformation was ΔΔG = –0.238 kcal/mol, suggesting that in the latter case the state was destabilized. However, when the variant was introduced into the active conformation of TLR8, the ΔΔG was +0.047 kcal/mol, indicating that the variant was predicted to be stabilizing the structure of the activated homodimer. Further, the analysis showed that changes in vibrational entropy energy were higher in the inactive state compared with the active state, suggesting that the active state conformation of the p.A518T variant was structurally more stable than its lesser stable inactive state (Figure 6C).

In addition to energetic calculations, 100-nanosecond molecular dynamics simulations were carried out with OpenMM (28) to investigate the dynamics of water-mediated hydrogen bonds of the WT and variant proteins across their active and inactive states. These simulations revealed that the number of water-mediated hydrogen bonds at the interface of the TLR8 homodimer increased in the A518T variant compared with the WT protein in the active state, across the entire length of the simulation (Figure 6D). Synchronous with the DynaMut predictions, the A518T variant in the inactivated state had fewer water-mediated interchain hydrogen bonds compared with the WT protein in the inactivated state. This selective increase in inter-protomer water bridging in the activated state of the A518T variant adds further support to the model that the A518T variant conferred a gain of function through an increase in water-mediated hydrogen bonding interactions.

Taken together, these energetic, structural, and dynamic modeling analyses suggested that the A518T variant stabilized the structure of the active state of the TLR8 homodimer, consistent with the inflammatory syndrome observed in the male siblings. These findings highlight the value of structural modeling in predicting the functional consequences of genetic mutations, and offer important insights into the molecular mechanisms underlying disease pathology.

### TLR8 A518T variant mediates gain of function following in vitro stimulation with TLR8 agonists.

**Figure 7:**
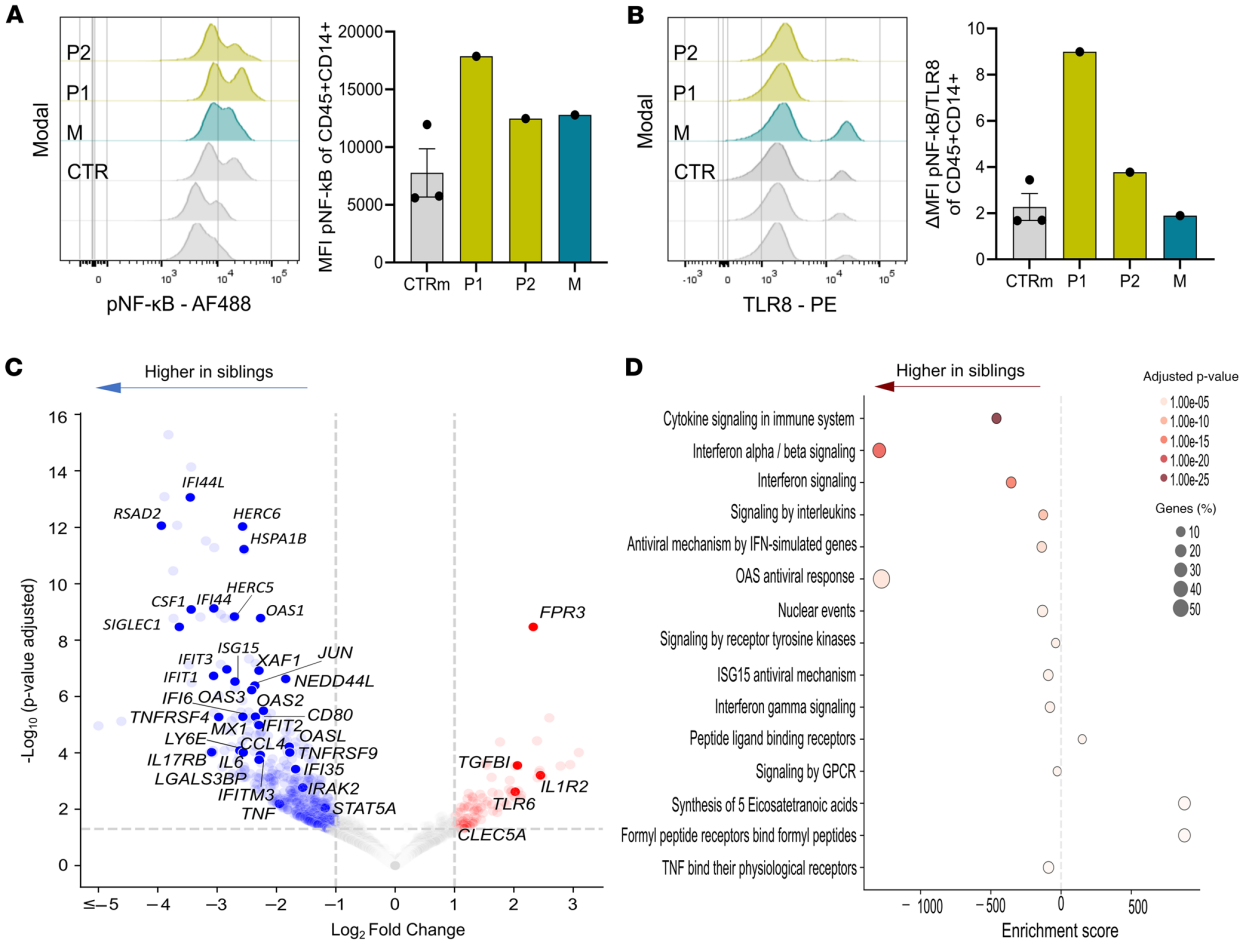
Functional studies demonstrate a gain of function of the TLR8 A518T variant. (**A**) Histogram plots of p–NF-κB of CD45^+^CD14^+^ monocytes from freshly isolated PBMCs in family members relative to healthy males (CTRm; *n* = 3) (left) with the corresponding MFI (right). Data represent mean ± SEM for healthy male controls. (**B**) Histogram plots of TLR8 of CD45^+^CD14^+^ monocytes from freshly isolated PBMCs in family members relative to healthy males (CTRm; *n* = 3) (left) and the ratio of ∆ p–NF-κB^+^ to TLR8^+^ of CD45^+^CD14^+^ monocytes (right). Data represent mean ± SEM for healthy male controls. (**C**) Volcano plot illustrating differentially expressed genes in unstimulated CD14^+^CD16^–^ monocytes isolated from healthy male controls (*n* = 4) compared with family members (P1 and P2). Genes with significant upregulation and downregulation are highlighted based on adjusted *P* values and fold change thresholds. (**D**) Bubble plot representing the top enriched pathways identified from the differentially expressed genes. Bubble size corresponds to the percentage of genes involved, and color intensity reflects the significance of pathway enrichment.

Given the predictions from the structural modeling suggesting enhanced structural stability of the TLR8 A518T variant in its active state, in vitro studies were performed to assess functional consequences. TLR8-mediated signaling through NF-κB induces the production of various proinflammatory cytokines (18). Flow cytometry analysis showed increased mean fluorescence intensity (MFI) of phosphorylated (p–) NF-κB p65 (Ser536) within unstimulated CD45^+^CD14^+^ monocytes of family members in comparison with healthy controls (Figure 7A). In contrast, P1 and P2 exhibited reduced TLR8 expression (MFI) in the same cell population, resulting in an overall elevated ratio of p–NF-κB to TLR8 MFI in CD45^+^CD14^+^ monocytes (Figure 7B). To further validate these findings of enhanced monocyte activation, we performed RNA sequencing analyses of enriched unstimulated CD14^+^CD16^–^ cells from family members and healthy male controls. Differential gene expression revealed upregulation of 515 genes in CD14^+^CD16^–^ cells of P1 and P2 compared with 98 genes in healthy control individuals. Several proinflammatory genes (*TNF*, *IL-6*, *CCL4*), ISGs (*OAS1*, *OAS2*, *MX1*, *IFIT1*, *IFIT3*, *IFI44L*), and genes commonly associated with M1 phenotype of macrophages, like *CD80*, as well as ubiquitin-related genes, including *HERC5*, *HERC6*, and *RNF144B*, were highly expressed in P1 and P2 compared with healthy control individuals (Figure 7C). Pathway analysis revealed enrichment of cytokine signaling, IFN signaling, and TNF-associated pathways in affected individuals compared with healthy control individuals (Figure 7D). Taken together, these data demonstrated enhanced expression of proinflammatory genes in unstimulated CD14^+^CD16^–^ cells of the affected siblings, consistent with the clinical presentation. Stimulation of CD14^+^CD16^–^ monocytes with 1 mg/mL TL8-506 for 4 hours resulted in upregulation of 55 genes in P1 and P2 compared with 9 genes in male controls. Among them were the transcription factors *JUN* and *FOS*, involved in macrophage activation, and *EGR1* and *KLF4*, participating in inflammatory processes; heat shock proteins such as *HSPA1B* and *HSPA6*; and ISGs like *IFIT2* (Supplemental Figure 10A). The most enriched pathways in P1 and P2 included cytokine and IFN signaling, as well as viral ribonucleoprotein (RNP) complex formation (Supplemental Figure 10B).

**Figure 8:**
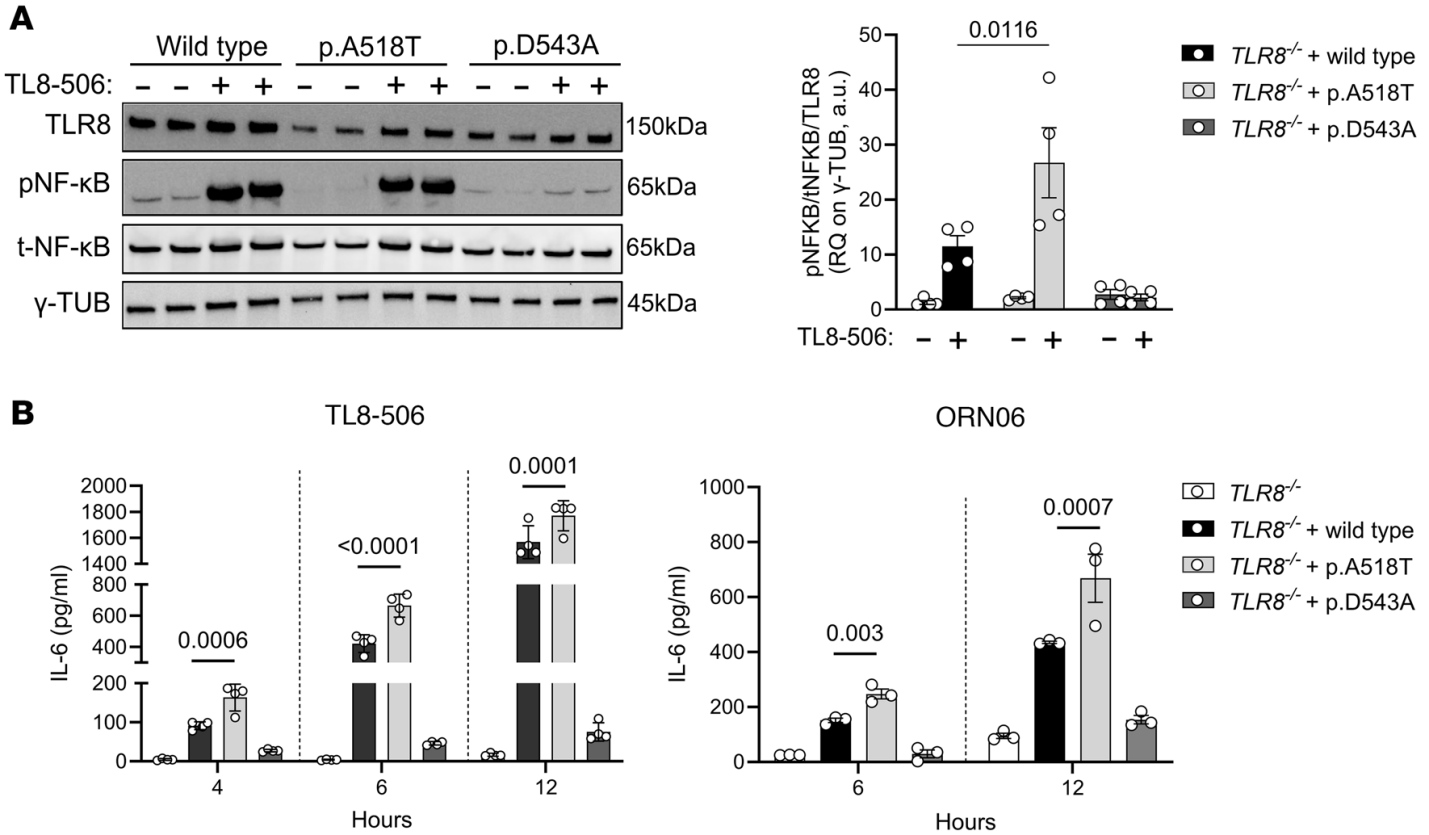
Stimulation of TLR8 A518T variant induces p–NF-κB and cytokine release. (**A**) Western blot against TLR8, p–NF-κB, total NF-κB, and γ-tubulin (γ-TUB) in BlaER1 *TLR8^–/–^* cells with the insertion of the TLR8 WT protein or the p.A518T and the previously published p.D543A (loss-of-function) TLR8 variants. Cells were stimulated with 100 ng/mL TL8-506 agonist for 15 minutes (left), and the ratio of p–NF-κB to total NF-κB to TLR8 levels, normalized to TLR8 levels, is shown (right). Statistical significance between groups was assessed using a 2-way ANOVA multiple-comparison test. Data represent mean ± SEM of *n* = 4 experiments. RQ, relative quantification. (**B**) IL-6 quantification of BlaER1 *TLR8^–/–^* cells with the insertion of the TLR8 WT protein or the p.A518T and p.D543A (loss-of-function) variants. Cells were stimulated with 1 μg/mL TL8-506 and 1 μg/mL ORN 06 agonists. Data are presented after the subtraction of the unstimulated measurements. Statistical significance between groups was assessed using a 2-way ANOVA multiple-comparison test. Data represent mean ± SEM of *n* *=* 3–4 experiments. Differences between groups were considered significant at *P* values less than 0.05.

To further elucidate the role of the TLR8 A518T variant in TLR8 responses, additional functional in vitro experiments were conducted using BlaER1, a human B cell precursor leukemia cell line that can be transdifferentiated into monocyte/macrophage–like cells. Using the PiggyBac transposon system (29), we generated BlaER1 *TLR8^–/–^* cells stably expressing either WT TLR8 protein or the p.A518T variant, along with the previously described gain-of-function variant (p.F494L) (5) and the loss-of-function variant (p.D543A) (30). Western blot analysis of protein extracts from BlaER1 *TLR8^–/–^* cells stimulated for 15 minutes with the TLR8 agonist TL8-506 (100 ng/mL) demonstrated increased p–NF-κB p65 (Ser536) in cells expressing the A518T variant relative to WT TLR8 protein (Figure 8A), indicating enhanced activation of downstream signaling. Interestingly, the F494L (gain-of-function) variant remained constitutively activated, as indicated by the presence of p–NF-κB already under unstimulated condition, along with an absence of total NF-κB (Supplemental Figure 11A).

To assess functional consequences for cytokine production, transdifferentiated BlaER1 *TLR8^–/–^* cells expressing either the TLR8 WT protein or p.A518T, p.F494L, and p.D543A TLR8 variants were stimulated with TLR8 agonists. Stimulation with either 1 μg/mL TL8-506 or 1 μg/mL ORN 06 (ssRNA with 6 UUGU repeats, requiring both binding sites for TLR8 activation) (12, 31, 32) led to a significant increase in IL-6 secretion in cells expressing the p.A518T variant compared with WT (Figure 8B), consistent with gain of function. Kinetic analyses showed that IL-6 secretion was detectable as early as 2 hours after stimulation with 50 ng/mL TL8-506 (data not shown), and after 4 hours with 100 ng/mL stimulation (Supplemental Figure 11B). TNF secretion was induced as early as 2 hours after stimulation with 100 ng/mL of the TLR8 agonist TL8-506 (Supplemental Figure 11C) and further increased after 4 hours of stimulation with both 50 and 100 ng/mL (Supplemental Figure 11D).

To further validate the functional impact of the TLR8 variants, HEK Blue Null 1 (HEK BN1) cells — lacking endogenous TLR8 — were transfected with TLR8 WT, p.A518T, and known gain-of-function variants (p.F492V and p.G572V), as well as the loss-of-function variant p.D543A, and stimulated with 100 ng/mL TL8-506. Stimulation with TL8-506 resulted in increased NF-κB reporter activity in cells expressing the p.A518T variant compared with WT (Supplemental Figure 11E). In contrast, stimulation with the TLR7-specific agonist CL097 (1 μg/mL) did not induce NF-κB activation in HEK BN1 cells transfected with the TLR8 p.A518T variant compared with cells transfected with the TLR8 p.F492V and p.G572V gain-of-function variants (Supplemental Figure 11F). Consistently, BlaER1 *TLR8^–/–^* cells reconstituted with TLR8 WT or the p.A518T variant did not produce IL-6 upon stimulation with CL097 (1 μg/mL; data not shown), indicating that TLR8 p.A518T signaling remained specific to TLR8 activation.

Taken together, these findings demonstrate that the TLR8 A518T variant confers a ligand-dependent gain-of-function phenotype characterized by enhanced NF-κB activation, upregulation of proinflammatory and IFN-stimulated genes, and increased secretion of key cytokines such as IL-6 and TNF upon TLR8, but not TLR7, agonist stimulation, in line with the inflammatory syndrome observed in the two siblings.

### Partial reduction of TLR8 protein abundance via proteasomal targeting in cells expressing the TLR8 A518T variant.

**Figure 9:**
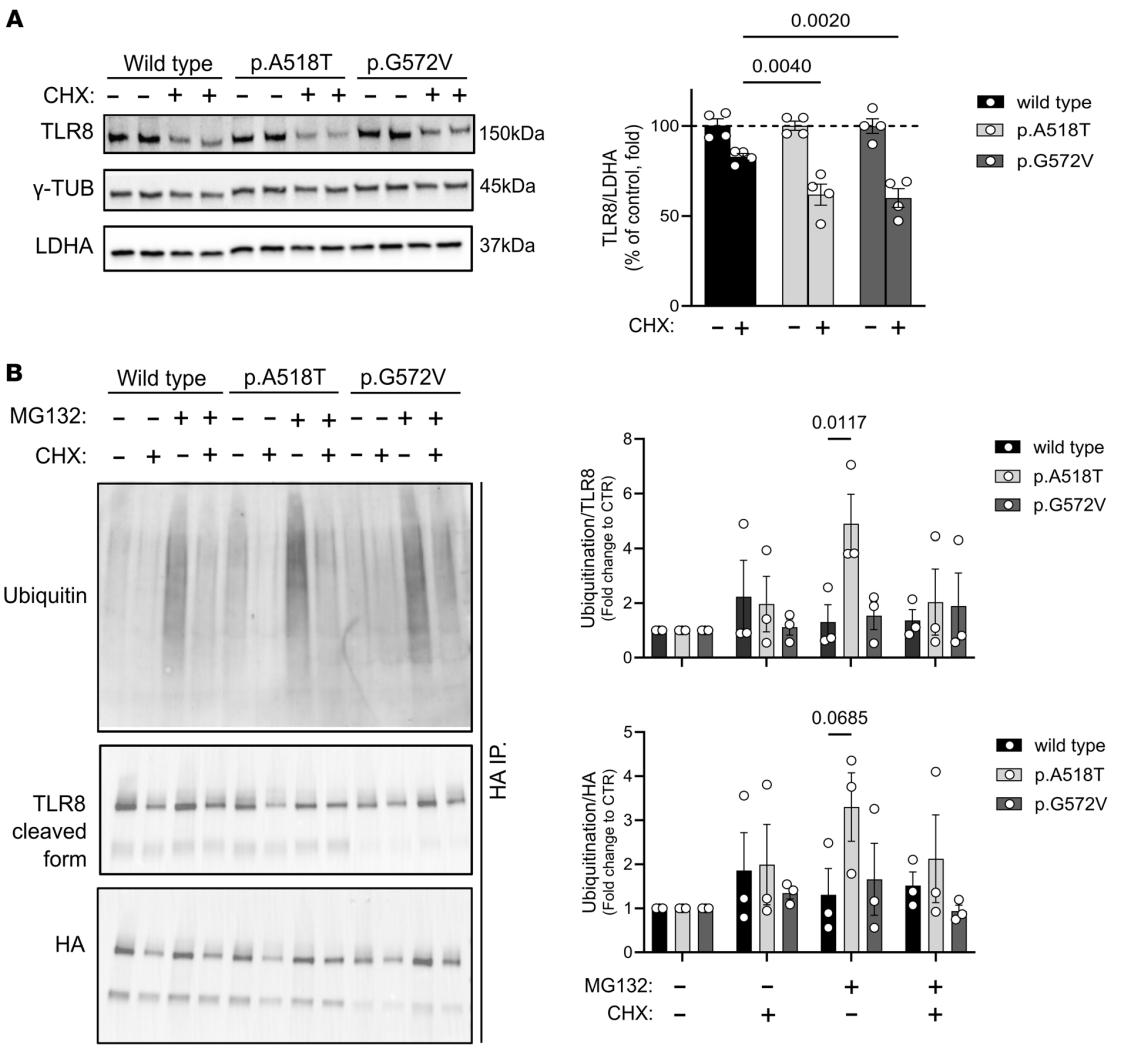
TLR8 A518T variant reduces protein abundance and increases degradation. (**A**) Western blot against TLR8, γ-TUB, and lactate dehydrogenase A (LDHA) of cycloheximide-incubated (CHX-incubated) HEK BN1 cells expressing TLR8 WT or the p.A518T and the previously published p.G572V (gain-of-function) variants (left), and Western blot quantification (right). Statistical significance between groups was assessed using a 2-way ANOVA multiple-comparison test. Data represent mean ± SEM of *n* = 4 experiments. (**B**) Western blot analysis of HA-tagged immunoprecipitated proteins from HEK293T cell extracts treated with (+) or without (–) the proteasome inhibitor MG132 and CHX. Cells were transfected with HA-tagged constructs and subjected to immunoprecipitation using anti-HA antibody. Left: Blots were probed with antibodies against ubiquitin, TLR8, and the HA tag to assess protein ubiquitination, TLR8 levels, and expression of the HA-tagged construct. Right: Quantification of ubiquitin signal normalized to TLR8 (top) and to HA (bottom) signals, showing the relative levels of TLR8 ubiquitination and overall ubiquitination of HA-tagged proteins. Statistical significance was assessed with a 2-way ANOVA multiple-comparison test. Data represent mean ± SEM of *n* = 3 experiments. Differences between groups were considered significant at *P* values less than 0.05.

While functional assays identified the TLR8 A518T variant as a gain-of-function mutation, Western blot analysis quantifying TLR8 protein levels also showed approximately 50% reduction in BlaER1 *TLR8^–/–^* cells expressing the p.A518T variant compared with those expressing TLR8 WT protein (Figure 8A and Supplemental Figure 12A), and these reduced levels persisted following stimulation with the 100 ng/mL TL8-506 agonist for 15 minutes (Supplemental Figure 12B). These findings suggest that the A518T variant leads to partial reduction in the abundance of the TLR8 protein. To investigate the potential mechanisms underlying reduced TLR8 A518T protein levels, additional protein turnover and degradation experiments were performed. For protein turnover experiments, HEK BN1 cells were transfected with plasmids encoding TLR8 WT, the p.A518T variant, or the known gain-of-function p.G572V variant (6). Forty-eight hours after transfection, cells were treated with cycloheximide (CHX) for 8 hours to inhibit new protein synthesis (33). Western blot analysis revealed that the TLR8 A518T variant underwent accelerated degradation compared with the TLR8 WT protein, with degradation levels comparable to those of the previously characterized G572V variant, which is known to exhibit faster protein turnover (Figure 9A).

Given that protein degradation primarily occurs via the ubiquitin-proteasome system, we next assessed the mechanism of degradation (34). HA-tagged TLR8 constructs were transfected into HEK293T cells and treated for 6 hours with CHX, the proteasome inhibitor MG132 (35), or both. HA-immunoprecipitation followed by Western blotting showed increased ubiquitination of the TLR8 A518T variant compared with TLR8 WT protein in the presence of MG132, indicating enhanced proteasomal targeting (Figure 9B). To further examine the proteasomal engagement, BlaER1 *TLR8^–/–^* cells expressing either TLR8 WT or p.A518T and p.D543A (loss-of-function) variants were treated with the proteasome inhibitor MG132 for 6 hours. Western blot analysis of cell extracts showed rescue of the protein levels for TLR8 p.A518T (Supplemental Figure 12C), further supporting the involvement of proteasomal degradation. Taken together, these results demonstrate that the TLR8 A518T variant undergoes accelerated degradation through increased ubiquitination and enhanced proteasomal targeting, consistent with the lower steady-state protein abundance of the TLR8 A518T variant.

## Discussion

Here, we describe a novel hemizygous TLR8 variant (c.1552G>A, p.A518T) identified in two male siblings with chronic inflammatory syndrome. Integration of genetic studies, computational modeling, and functional ex vivo and in vitro analyses revealed a complex immunological phenotype in male carriers of the TLR8 p.A518T variant characterized by enhanced TLR8-mediated NF-κB signaling with elevated proinflammatory cytokine profiles, and profound immune cell dysregulation, including significant B cell abnormalities.

Gain-of-function variants in the *TLR8* gene have been described to cause hyperactivation of TLR8 upon ligand recognition, leading to overproduction of inflammatory cytokines (5, 6), and are associated with B cell impairment (5). These variants contribute to a spectrum of immune disorders (2, 7, 36), including a recent gain-of-function variant in TLR8 that has been implicated in impaired erythropoiesis (8). Notably, pathogenic roles of TLR8 have been described in rheumatoid arthritis (37), antiphospholipid syndrome (38, 39), and HIV disease susceptibility (40). Unlike previous reports where low-level mosaicism led to symptoms later in life, the present cases showed early onset and severe symptoms in both individuals, consistent with a germline variant (5). The two male siblings presented with recurrent infections and severe inflammatory syndrome in the first months of life, while peripheral blood analysis showed increased NF-κB and IFN signaling in comparison with healthy control individuals, consistent with previously reported cases (5).

TLR8 is predominantly expressed in macrophages, dendritic cells, and neutrophils. We observed classical monocyte frequencies comparable to those of healthy controls; however, transcriptomic profiles of monocytes showed a heightened immune activation at baseline and upon stimulation, with upregulation of NF-κB and IFN pathways and increased expression of genes involved in ubiquitin-proteasome degradation. Additionally, an increased frequency of intermediate monocytes and a reduction in non-classical monocytes were observed exclusively in P2, with the decrease in non-classical monocytes aligning with findings from previous studies (5). Within dendritic cells, reduced frequencies of CD141^+^ cells and plasmacytoid dendritic cells were noted, the latter having been also reported previously (6). IL-6 and TNF are key cytokines involved in CD4^+^ T cell exhaustion — present in both siblings — particularly in the context of chronic inflammation and persistent infection (41). Previous studies have identified IL-6 signaling as a major early inducer of Tfh differentiation (42), and TLR8 activation has been shown to enhance Tfh function (43, 44), playing an important role in supporting B cell differentiation into memory B cells and antibody-secreting plasmablasts. In line with the described role of IL-6 in Tfh cell activation, we observed an expansion of Tfh cells, along with the expression of the cell surface markers ICOS and CD40L in both siblings. However, this Tfh cell expansion coincided with a marked delay in activation of naive B cells and B cell maturation, characterized by a complete loss of memory B cells and plasma cells in both carriers, a finding that was also observed in a previous study (5). This immunological profile manifested clinically with delayed IgG and IgM production and suboptimal vaccine responses, highlighting a significant impairment in humoral immunity.

Detailed immunological studies revealed clear differences between the siblings at the time of analysis, which may be attributed to variations in disease progression, infection status, or medication. Additionally, the heterogeneity in T cell subsets might also be attributed to the presence of a second mutation in the *DNAJC21* gene (c.–171del), which was unique to P2. DNAJC21-related diseases constitute a distinct inherited bone marrow failure syndrome with features that overlap with Schwachman-Diamond syndrome (45). Previously reported mutations in the *DNAJC21* gene have been associated with a spectrum of bone marrow failure phenotypes (45–47), which may explain the significantly lower lymphocyte counts observed in P2. Collectively, our immunological findings highlight the immunological profile associated with a germline variant of TLR8, characterized by alterations in myeloid cell populations and significant impairment of B cells. While targeted sequencing enabled the identification of the causative TLR8 variant in this family, broader genomic approaches — such as whole-exome or whole-genome sequencing — might provide a more comprehensive assessment of additional genetic contributors in the future, especially in complex cases with variable clinical presentations, as observed in P2.

In vitro functional assays in BlaER1 *TLR8^–/–^* cells with genome-integrated WT or mutant TLR8 also demonstrated enhanced immune cell activation, evidenced by increased p–NF-κB levels and elevated IL-6 and TNF production upon TLR8 stimulation. Interestingly, both TLR8 ligands that bind to site 1 (located at the apex of the dimerization interface and binding uridine molecules, including synthetic TLR8 agonists such as TL8-506) (31, 32) and site 2 (which binds short oligo-ribonucleotides) (12) were capable of activating the TLR8 A518T variant to a greater degree compared with the WT protein, confirming gain of function. Additional functional assays revealed reduced protein availability of the TLR8 A518T variant due to accelerated degradation, potentially mediated by ubiquitination and enhanced targeting to the proteasomal pathway. Complementary computational analyses showed that, in the active state, the A518T substitution increases the number of water-mediated hydrogen bonds at the structure of homodimer interface, compared with TLR8 WT receptor. This structural rearrangement stabilized the structure of the active conformation of the TLR8 homodimer, in line with enhanced receptor signaling upon stimulation. These findings suggest two consequences of the TLR8 A518T variant: a ligand-dependent gain of function leading to heightened inflammatory signaling, alongside reduced protein abundance resulting from accelerated protein turnover and degradation. Together, these mechanisms shaped the complex immunopathology observed clinically in both siblings.

Importantly, our study demonstrates the utility of integrating computational structural modeling with functional assays as a complementary strategy to support the interpretation of rare genetic variants. Such combined approaches have the potential to accelerate diagnosis and guide personalized treatment strategies, especially in rare immunological disorders where clinical symptoms and genetic data can be complex and heterogeneous. Given the increasing recognition of TLRs as key players in IEI, autoimmune, and autoinflammatory diseases, our findings emphasize the promise of precision immunomodulatory therapies tailored to individual genetic alterations affecting innate immune pathways.

In conclusion, the TLR8 c.1552G>A (p.A518T) missense variant stabilized the active structure of the receptor homodimer and conferred a ligand-dependent gain of function upon receptor activation, enhancing downstream immune activation and driving a pronounced hyperinflammatory state, despite reduced protein abundance. Our findings broaden the clinical and molecular spectrum of TLR8-associated immune disorders and underscore the value of integrated genetic, structural, and functional approaches in elucidating the pathogenicity of rare innate immune variants.

## Methods

### Sex as a biological variable.

Our study exclusively examined two male siblings and a female, their mother, because the disease studied is relevant to the X chromosome.

### Immunophenotyping of PBMCs by multiparametric flow cytometry analyses.

Immunophenotyping was performed in freshly isolated or frozen PBMCs from blood of the family members (P1, P2, and M) and age-matched healthy controls with age range from 20 to 30 years old (*n* = 3 men, *n* = 3 women). Frozen PBMCs were thawed before staining. All antibodies used in the study are listed in Supplemental Table 2. Detailed methodology is provided in Supplemental Methods.

### Computational protein modeling.

The 3-dimensional structures of the active-state (liganded) (PDB ID: 4R08) (12) and inactive-state (unliganded) (PDB ID: 3W3G) (16) conformations of the human TLR8 receptor were obtained from the Protein Data Bank (PDB) (48). The structure of the A518T variant was predicted using homology modeling with MODELLER v10.3 (26). The effect of the A518T mutation on the activated and inactivated states of TLR8 was analyzed with DynaMut (27). The hydrogen bond interactions at the interface of the TLR8 dimer were calculated and visualized using UCSF ChimeraX v1.7 (49). All molecular dynamics simulations were performed using OpenMM 8.1.2 (28). Initial structures were completed with MODELLER v10.3. Ligand parameters were generated using the tleap module from the Amber suite (50). Simulations were conducted for 4 systems: TLR8 WT in active and inactive states, and the A518T variant in both conformations. Each system was first minimized using a Verlet integrator with a 1-femtosecond time step and particle mesh Ewald electrostatics. Energy minimization was carried out with a convergence criterion of 1.0 kJ/mol/nm over a maximum of 500 iterations. After minimization, a 2-step equilibration protocol was used: NVT (canonical ensemble) equilibration at 300K for 500 picoseconds using a Langevin integrator with a 2-femtosecond time step and friction coefficient of 1 picosecond^–1^ and NPT (isothermal-isobaric ensemble) equilibration for 500 picoseconds under 1 bar pressure using a Monte Carlo barostat and the same Langevin integrator setup. Production simulations were run for 100 nanoseconds (50,000,000 steps at 2 femtoseconds) in the NPT. Coordinates were saved every 2 nanoseconds, and thermodynamic properties were monitored throughout the trajectory. We evaluated the water-mediated hydrogen bonds at the interface using an in-house Python script with distance cutoff of 3.5 Å and angle cutoff of 150°.

### Cell culture.

Malignant B-lineage BLaER1 *TLR8^–/–^* cells were used for functional experiments. BLaER1 *TLR8^–/–^* cells with TLR8 WT protein or TLR8 p.A518T and F494L (gain of function), D543A (loss of function) (16), were generated with the PiggyBac transposon system (29), as described in Supplemental Methods. BLaER1 cells were cultivated in RPMI 1640 medium supplemented with l-glutamine (11835-030, Gibco), 1 mM sodium pyruvate (S83636, Sigma-Aldrich), 10% heat-inactivated FBS (FBS-HI-11F, Capricorn), and 1% penicillin-streptomycin. BLaER1 cells were transdifferentiated into monocytes/macrophages for 5 days in medium containing 10 ng/mL of recombinant human IL-3 (203-IL-010/CF, R&D Systems), 10 ng/mL recombinant human M-CSF (574804, BioLegend), and 100 nM β-estradiol (E8875, Sigma-Aldrich) at 8 × 10^4^ cells per well of 96-well plates. Twenty-four hours before stimulation, the cells were stimulated with fresh medium containing 0.5 μg/mL doxycycline hyclate (D5207, Sigma-Aldrich) (51). Cells were then stimulated with either TLR8 agonists — 50 ng/mL, 100 ng/mL, or 1 μg/mL TL8-506 (tlrl-tl8506, InvivoGen) and 1 μg/mL ORN 06 (tlrl-orn6, InvivoGen) — or 1 μg/mL CL097 TLR7 agonist. In some experiments cells were treated with MG132 (10 μM; M7449-200UL, Merck) to block proteasomal degradation. Cell extracts or supernatants were collected and stored for subsequent analyses.

For NF-κB activity experiments, HEK Blue Null 1 (HEK BN1, hkb-null1, InvivoGen) cells were seeded at a concentration of 7.5 × 10^4^ into each well of 96-well plates and incubated at 37°C for 24 hours with DMEM high glucose (11965092, Gibco) supplemented with 10% FBS (Capricorn), 1% penicillin-streptomycin. The medium was further supplemented with additional antibiotics according to the manufacturer’s instructions. Cells were transfected with 130 ng of plasmid DNA encoding either TLR8 WT or one of the variants (p.A518T, p.F494L, p.G572V, or p.D543A) using the jetOPTIMUS DNA transfection reagent (101000006, Polyplus-transfection SA) according to the manufacturer’s instructions. Medium was changed to fresh plating medium after 5 hours of incubation at 37°C. Cell culture supernatants were collected and used for secreted embryonic alkaline phosphatase assay.

For the cycloheximide chase assay and the HA-tagged immunoprecipitation/ubiquitination experiments, HEK BN1 and HEK293T cells were used, respectively. Cells were seeded at a concentration of 4 × 10^5^ into each well of 6-well plates and incubated at 37°C for 24 hours with DMEM high glucose (11965092, Gibco) supplemented with 10% FBS (Capricorn), 1% penicillin-streptomycin. The medium for the HEK BN1 cells was further supplemented with additional antibiotics according to the manufacturer’s instructions. Cells were then transfected using jetOPTIMUS DNA transfection reagent (Polyplus-transfection SA) according to the manufacturer’s protocol. Cells were transfected with 2 μg of plasmid DNA encoding either TLR8 WT or one of the variants (p.A518T, p.G572V). Medium was changed to fresh plating medium after 5 hours of incubation at 37°C.

### Statistics.

Normal distribution of the data was assessed using the Shapiro-Wilk normality test. Comparative statistics between normally distributed groups were calculated using Student’s 2-tailed *t* test and 1- or 2-way ANOVA multiple-comparison test. Differences between groups were considered significant at *P* values less than 0.05. All statistical analyses were performed with GraphPad Prism 10.0 (GraphPad Software).

### Study approval.

Healthy participants, including age-matched (to siblings and mother) men and women, were recruited at the University Hospital Hamburg-Eppendorf. The study received approval from the Ethical Committee of the medical association of the Freie und Hansestadt Hamburg (Ärztekammer Hamburg) under ethical permit PV4780. The study followed the guidelines of the Declaration of Helsinki. Before enrollment in the study, each participant provided written informed consent.

For family participants, the study received approval from the Ethical Committee of the medical associations of the Freie und Hansestadt Hamburg (Ärztekammer Hamburg) and Schleswig-Holstein (Ärztekammer Schleswig-Holstein) under permits 024-101307-BO-ff and 041/24 m (BO), respectively. The study followed the guidelines of the Declaration of Helsinki. Before enrollment in the study, each participant provided informed consent. The guardian and participants provided written informed consent. Blood samples were collected from participants (P1 and P2) and the guardian (mother, M).

### Data availability.

All data are available in the main text or the supplemental materials. The corresponding author will respond to emailed requests to share additional methods and data. Additional detailed methodology can be found in Supplemental Methods.

## Author contributions

We applied the sequence-determines-credit (SDC) approach for the sequence among the first 4 coauthors.

NTS, EL, IK, and MA conceptualized the study. NTS, EL, SN, IE, MP, ME, FH, MB, BP, FJF, JMC, BG, AS, BB, SB, PB, KR, HH, UM, SGJ, JH, VON, MK, ML, and FRS developed methodology. NTS, EL, SN, IE, MP, ME, FH, MB, BP, FJF, JMC, BG, AS, BB, SB, PB, KR, HH, UM, SGJ, JH, VON, MK, CK, FKN, NMT, ET, ML, FRS, VH, MJB, CS, MT, IK, and MA performed investigation. NTS, SN, IE, ME, FH, FJF, AS, BB, SB, and KR performed visualization. VH, CS, MT, and MA acquired funding. NTS, IK, and MA performed project administration. MA supervised the study. NTS, EL, SN, IE, ME, FH, MB, BP, PB, CK, and MA wrote the original draft of the manuscript. NTS, EL, SN, IE, MP, ME, FH, MB, BP, FJF, JMC, BG, AS, BB, SB, PB, KR, HH, UM, SGJ, JH, VON, MK, CK, FKN, NMT, ET, ML, FRS, VH, MJB, CS, MT, IK, and MA reviewed and edited the manuscript.

## Funding support

- Deutsche Forschungsgemeinschaft (DFG) Research Unit 5068, Sex Differences in Immunity (429191104).
- Cooperation of the Leibniz Institute of Virology Strategic Incentive Program.
- The Leibniz ScienceCampus InterACt (funded by the Leibniz Association and the Ministry of Science, Research, Equality and Districts [BWFGB] of the City of Hamburg, as well as by the Leibniz Institute of Virology and the Universität Hamburg).
- Landesforschungsförderung Hamburg (LFF FV-UHH-09).
- DFG CRC1648.
- German Federal Ministry of Education and Research (BMBF) Computational Life Sciences project (ASPIRE, 031L028).
- German Center for Child and Adolescent Health (DZKJ), a partner site in Munich (to VH).
- DFG 450149205-TRR333/1 and SCHL2276/2-1 (to CS).
- Daisy Huet Roell Foundation (DHRF).

## Supplementary Material

Supplemental data

Unedited blot and gel images

undefined

## Figures and Tables

**Table 1:**
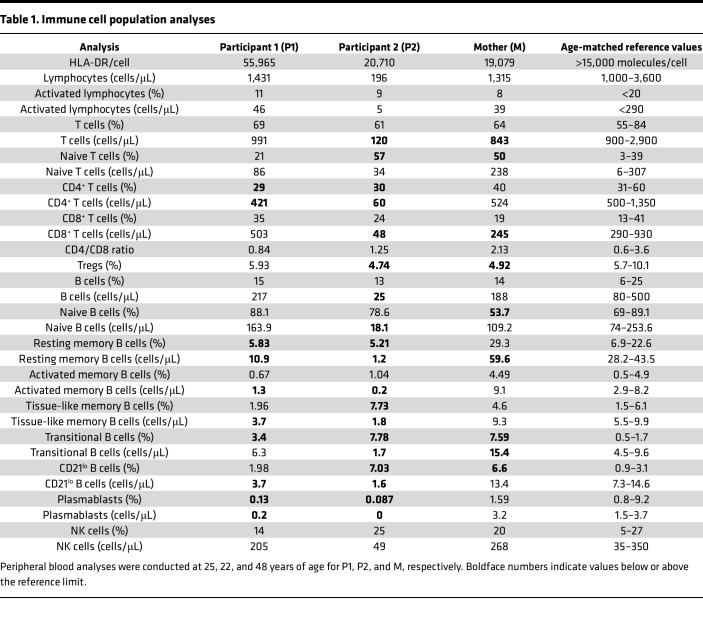
Immune cell population analyses

**Table 2:**
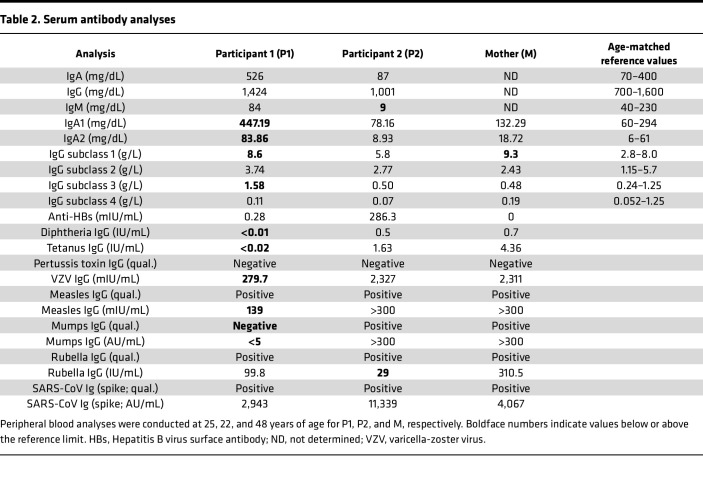
Serum antibody analyses

**Table 3:**
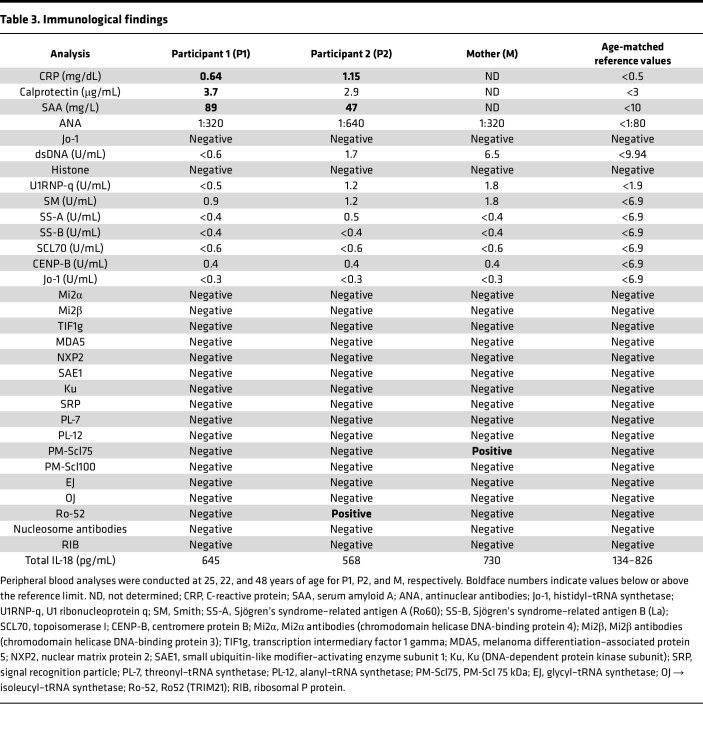
Immunological findings

## Version 1 02/23/2026

Electronic publication

## Version 2 03/02/2026

Improved PDF resolution

## References

[1] Tangye SG (2022). Human inborn errors of immunity: 2022 update on the classification from the International Union of Immunological Societies Expert Committee. J Clin Immunol.

[2] Akalu YT, Bogunovic D (2024). Inborn errors of immunity: an expanding universe of disease and genetic architecture. Nat Rev Genet.

[3] Cheng J (2023). Accurate proteome-wide missense variant effect prediction with AlphaMissense. Science.

[4] Li C (2024). Structural and functional prediction, evaluation, and validation in the post-sequencing era. Comput Struct Biotechnol J.

[5] Aluri J (2021). Immunodeficiency and bone marrow failure with mosaic and germline TLR8 gain of function. Blood.

[6] Fejtkova M (2022). TLR8/TLR7 dysregulation due to a novel TLR8 mutation causes severe autoimmune hemolytic anemia and autoinflammation in identical twins. Am J Hematol.

[7] Bleesing J (2022). Gain-of-function defects in toll-like receptor 8 shed light on the interface between immune system and bone marrow failure disorders. Front Immunol.

[8] Liang J (2024). Erythroid-intrinsic activation of TLR8 impairs erythropoiesis in inherited anemia. Nat Commun.

[9] Bauernfeind F (2010). An unexpected role for RNA in the recognition of DNA by the innate immune system. RNA Biol.

[10] Sarvestani ST (2012). Human Toll-like receptor 8 can be cool too: implications for foreign RNA sensing. J Interferon Cytokine Res.

[11] Ohto U (2014). Structure and function of toll-like receptor 8. Microbes Infect.

[12] Tanji H (2015). Toll-like receptor 8 senses degradation products of single-stranded RNA. Nat Struct Mol Biol.

[13] Chen X (2013). microRNAs are ligands of Toll-like receptors. RNA.

[14] Vollmer J (2005). Immune stimulation mediated by autoantigen binding sites within small nuclear RNAs involves Toll-like receptors 7 and 8. J Exp Med.

[15] Guiducci C (2013). RNA recognition by human TLR8 can lead to autoimmune inflammation. J Exp Med.

[16] Tanji H (2013). Structural reorganization of the Toll-like receptor 8 dimer induced by agonistic ligands. Science.

[17] Kawai T, Akira S (2011). Toll-like receptors and their crosstalk with other innate receptors in infection and immunity. Immunity.

[18] Cervantes JL (2012). TLR8: the forgotten relative revindicated. Cell Mol Immunol.

[19] Bender AT (2020). TLR7 and TLR8 differentially activate the IRF and NF-κB pathways in specific cell types to promote inflammation. Immunohorizons.

[20] Karczewski KJ (2020). The mutational constraint spectrum quantified from variation in 141,456 humans. Nature.

[21] Schubach M (2024). CADD v1.7: using protein language models, regulatory CNNs and other nucleotide-level scores to improve genome-wide variant predictions. Nucleic Acids Res.

[22] Adzhubei IA (2010). A method and server for predicting damaging missense mutations. Nat Methods.

[23] Fadra N (2024). Identification of skewed X chromosome inactivation using exome and transcriptome sequencing in patients with suspected rare genetic disease. BMC Genomics.

[24] Cain D (2009). Effects of acute and chronic inflammation on B-cell development and differentiation. J Invest Dermatol.

[25] Tangye SG (2023). Inborn errors of human B cell development, differentiation, and function. J Exp Med.

[26] Šali A (1993). Comparative protein modelling by satisfaction of spatial restraints. J Mol Biol.

[27] Rodrigues CHM (2018). DynaMut: predicting the impact of mutations on protein conformation, flexibility and stability. Nucleic Acids Res.

[28] Eastman P (2024). OpenMM 8: molecular dynamics simulation with machine learning potentials. J Phys Chem B.

[29] Zhao S (2016). PiggyBac transposon vectors: the tools of the human gene encoding. Transl Lung Cancer Res.

[30] Gibbard RJ (2006). Conserved features in the extracellular domain of human toll-like receptor 8 are essential for pH-dependent signaling. J Biol Chem.

[31] Forsbach A (2008). Identification of RNA sequence motifs stimulating sequence-specific TLR8-dependent immune responses. J Immunol.

[32] Greulich W (2019). TLR8 is a sensor of RNase T2 degradation products. Cell.

[33] Miao Y (2023). Cycloheximide (CHX) chase assay to examine protein half-life. Bio Protoc.

[34] Zhao L (2022). Targeted protein degradation: mechanisms, strategies and application. Signal Transduct Target Ther.

[35] Worthmann A (2024). Fatty acid synthesis suppresses dietary polyunsaturated fatty acid use. Nat Commun.

[36] Aluri J, Cooper MA (2023). Somatic mosaicism in inborn errors of immunity: current knowledge, challenges, and future perspectives. Semin Immunol.

[37] Sacre SM (2008). Inhibitors of TLR8 reduce TNF production from human rheumatoid synovial membrane cultures. J Immunol.

[38] Döring Y (2010). Human antiphospholipid antibodies induce TNFalpha in monocytes via Toll-like receptor 8. Immunobiology.

[39] Prinz N (2011). Antiphospholipid antibodies induce translocation of TLR7 and TLR8 to the endosome in human monocytes and plasmacytoid dendritic cells. Blood.

[40] Oh DY (2008). A functional toll-like receptor 8 variant is associated with HIV disease restriction. J Infect Dis.

[41] Alahdal M, Elkord E (2022). Exhaustion and over-activation of immune cells in COVID-19: challenges and therapeutic opportunities. Clin Immunol.

[42] Choi YS (2013). Cutting edge: STAT1 is required for IL-6-mediated Bcl6 induction for early follicular helper cell differentiation. J Immunol.

[43] Ayithan N (2021). Follicular helper T (TFH) cell targeting by TLR8 signaling for improving HBsAg-specific B cell response in chronic hepatitis B patients. Front Immunol.

[44] Ugolini M (2018). Recognition of microbial viability via TLR8 drives T_FH_ cell differentiation and vaccine responses. Nat Immunol.

[45] D’Amours G (2018). Refining the phenotype associated with biallelic DNAJC21 mutations. Clin Genet.

[46] Alsavaf MB (2022). A novel missense mutation outside the DNAJ domain of DNAJC21 is associated with Shwachman-Diamond syndrome. Br J Haematol.

[47] Chirita-Emandi A (2022). Case report: novel biallelic variants in DNAJC21 causing an inherited bone marrow failure spectrum phenotype: an odyssey to diagnosis. Front Genet.

[48] Berman HM (2000). The Protein Data Bank. Nucleic Acids Res.

[49] Meng EC (2023). UCSF ChimeraX: tools for structure building and analysis. Protein Sci.

[50] Case DA (2023). AmberTools. J Chem Inf Model.

[51] Gaidt MM (2018). Modeling primary human monocytes with the trans-differentiation cell line BLaER1. Methods Mol Biol.

