# Supplementary material for: Structural modeling and functional characterization of a novel gain-of-function TLR8 variant causing severe inflammatory syndrome
Source: JCI Insight. 2026 Feb 23;11(4):e187422. doi: 10.1172/jci.insight.187422 (PMC12956005; doi:10.1172/jci.insight.187422)
Supplement: Supplemental data [file jciinsight-11-187422-s276.pdf]

Supplemental material

Structural modeling and functional characterization of a novel gain-of-function TLR8 variant causing severe inflammatory syndrome

Supplemental Methods

Genetic test

Next-generation sequencing (NGS) analysis was conducted using exome enrichment from DNA isolated from peripheral blood mononuclear cells (PBMCs) of the family members. Based on clinical information, our focus was formally directed towards genes including *ADA2*, *STING1*, *TNFAIP3*, *ARPC1B*, *NFKB1*, *SBDS*, *SRP54*, *EFL1*, *DNAJC21*, *C2orf69*, *CDC42* and *TLR8* as well as other genes relevant for differential diagnosis.

X-chromosome inactivation analysis

Peripheral blood mononuclear cells (PBMCs) were isolated from the mother by standard density gradient centrifugation. Genomic DNA was extracted and X-chromosome inactivation (XCI) patterns were assessed by quantitative fluorescent PCR (QF-PCR) analysis targeting two polymorphic, X-linked STR loci located in the androgen receptor gene (AR) and the retinitis pigmentosa 2 gene (RP2), respectively. These loci were chosen due to adjacent sites for the methylation-sensitive restriction enzyme *HpaII*, enabling discrimination of active and inactive X chromosomes. Following digestion with *HpaII*, QF-PCR amplification was performed with locus-specific primers and digested and undigested DNA samples, respectively (primer sequences available upon request). The relative amplification of each allele was quantified, and XCI ratios were calculated as the proportion of each allele's signal

compared to total. Ratios near 0.5 indicate random XCI, while deviations suggest skewing. The analysis was done at the Institute of Human Genetics, University Medical Center Hamburg-Eppendorf.

Isolation and freezing of PBMCs

Blood from study's subjects was drawn in EDTA tubes and the processing of the blood was initiated within 90 minutes after venipuncture. PBMCs were isolated using Lymphocyte Separation Medium (#LSM-A; Capricorn Scientific) density gradient centrifugation. Remaining erythrocytes were lysed in 3 mL ACK Lysing Buffer (#A10492-01, Gibco) for 3 minutes. Isolated PBMCs were re-suspended in RPMI Medium 1640 supplemented with glutamine (#21875091, Gibco), 10% heat inactivated fetal bovine serum (FBS, # FBS-11A, Capricorn) and 1% penicillin, streptomycin. Freshly isolated PBMCs frozen in FBS with 10% DMSO (#D5879, Sigma-Aldrich) at -80 ºC and then transferred the next day into liquid nitrogen.

Immunophenotyping of PBMCs by multiparemetric flow cytometry analyses

Briefly, PBMCs were collected and washed once with warm PBS. Cells were resuspended in master mix solution containing PBS, the appropriate antibodies (listed in Supplemental Table 2) and the live/dead stain near infrared (NIR) dye and incubated at 4 °C in the dark for 30 minutes. After a washing step with 2% FBS in PBS, cells were fixed/permeabilized for intracellular staining, with 150 µL of BD Cytofix/Cytoperm™ Fixation/Permeabilization Kit (#554714, BD Biosciences) at 4 °C for 20 minutes in the dark. After a washing step with 1X BD Perm/Wash™ Buffer, cells were stained with intracellular antibodies for 30 minutes at 4 °C in the dark. After a washing step with 2% FBS in PBS, cells were resuspended in 200 µL 2% FBS in PBS. In some staining protocols, intracellular TLR8 staining was performed on fixed cells with 4% paraformaldehyde for 10 minutes at room temperature and permeabilized with 0.1% Triton X. For phosphoflow assays, cells were fixed in 4% paraformaldehyde for 10 minutes at room temperature, then resuspended in 100% cold methanol, and incubated for 30 minutes on ice. Cells were then stained overnight with anti-pNF-κB p65 antibody (Ser536). Flow cytometry analysis was performed using a 5-laser Cytek Aurora Flow Cytometer (Cytek Biosciences). Resulting unmixed FCS files were analyzed with FlowJo v10.5 software (BD Life Sciences). Freshly

isolated blood was independently analyzed at the Institute of Immunology, University Medical Center Hamburg-Eppendorf, in order to quantify the absolute and relative cell population numbers.

Stimulation of B cells

Negative selection of B cells from PBMCs was conducted using the EasySep™ Human Pan-B Cell Enrichment Kit (#19554, StemCell). B cells were resuspended at a density of 0,5 x 10^5 cells/ml in Iscove's Modified Dulbecco's Medium (IMDM, Gibco) containing 10% FBS (Gibco), 50µM β-Mercaptoethanol (Gibco), 2 mM L-Glutamine (Gibco), 1 mM Sodium-Pyruvate (Gibco), 0.1 mM NEAA (Gibco), 10 mM HEPES (Gibco), 100 µg/ml streptomycine with 100 U/ml Penicillin (Gibco), 100 U/ml IL-2 (Miltenyi Biotec), 0.05 µg/ml IL-21 (Gibco). Stimulation of B cells was performed using the Human CD40-Ligand Multimer Kit by Miltenyi Biotec (#130-098-776, Miltenyi Biotec). Cells were seeded on a 24 well plate and incubated for 7 days at 37 °C, 5% CO_2. B cells and supernatant were collected on day 0, 2, 4 and 7 for further analyses.

Immunophenotyping of stimulated B cells

B cells were washed with 0.2% BSA in PBS and incubated with 1 mg/ml purified human IgG (Jackson Immuno Research) at a dilution of 1:100 for 10 minutes at 4 °C. Afterwards, B cells were stained with fluorochrome-conjugated antibodies for 20 minutes at 4 °C and washed twice. The primary antibodies used for staining included: CD45-BV510 (HI30, Biolegend), CD19-BV785 (HIB19, Biolegend), CD38-FITC (HIT2, Biolegend), CD20-BB700 (2H7, BD Biosciences), CD27-PE (LG.3A10, Biolegend), IgD-APC (IA6-2, Biolegend). Dead cells were detected with Alexa Fluor 750 NHS Ester (#A20011, Invitrogen). Flow cytometry measurement was performed on BD FacsSymphony A1. Resulting unmixed FCS files were analyzed with FlowJo v10.5 software (BD Life Sciences).

CD4+ T cell stimulation

PBMCs were thawed in complete, pre-warmed medium of RPMI 1640 (#21875091, Gibco) supplemented with 10% FBS (Capricorn). Cells were washed and resuspended in autoMACS Running Buffer – MACS Separation Buffer (#130-091-221, Miltenyi Biotec). CD4+ T cells were negatively enriched from PBMCs using an EasySep™ Negative Human CD4 Kit (#19052, StemCell) following

manufacturer recommendations. CD4+ T cells were subsequently counted and resuspended in a 96 well plate U-bottom in complete medium with either 100U/mL IL-2 (#200-02-500UG, Peprotech) only or activated with 100U/mL IL-2 plus 25µl/mL Immunocult CD3/CD28 (#10991, StemCell). Cells at baseline (Day0) and activated cells (Day3) were stained using the same protocol. Briefly, cells were resuspended in a master mix containing LIVE/DEADTM Fixable Near-IR Dead (ThermoFisher) and the following antibodies antiCD3-BUV496 (clone UCHT1), antiCD27-BV661 (clone L128), antiCD4-BV785 (clone RPA-T4), antiCD8-R718 (clone HIT8), antiPD-1-BV605 (clone NAT105), and antiCXCR5-BV750 (clone RF8B2). The catalog number of the antibodies are listed in the Supplemental Table 2. Cells were incubated with the antibody mix for 20 minutes at room temperature, washed with autoMACS Running Buffer, fixed with 4% paraformaldehyde for 20 minutes at room temperature, washed and resuspended in autoMACS Running Buffer before flow cytometric acquisition. Flow cytometry analysis was performed using a 5-laser Cytek Aurora Flow Cytometer (Cytek Biosciences). Resulting unmixed FCS files were analyzed with FlowJo v10.5 software (BD Life Sciences).

CD14+ isolation and RNA isolation for RNA sequencing

CD14+CD16- monocytes were negatively isolated with EasySep™ Human Monocyte Enrichment Kit (#19059, StemCell), according to the manufacturer’s protocol. Trizol-resuspended monocytes were thawed on ice and chloroform was added to extract the RNA from the cells. To precipitate the RNA, Zymo Research Direct-zol RNA Microprep extraction kit was used (#R2062, Zymo Research). RNA integrity was analyzed by applying a TapeStation High Sensititvity RNA ScreenTape assay (5067-5579, 5067-5580, 5067-5581, Agilent). Per sample, 10 µl total RNA was Poly(A)-captured applying the Lexogen Poly(A) RNA Selection Kit and further processed via the RNA-Seq V2 Library Prep Kit with UDIs (#181.96, Lexogen) in the short insert size variant (RTM) according to the manufacturer’s instructions (applying 15 cycles of Library Amplification PCR, step 4.3, User Guide version 171UG394V0111).

Libraries were quality controlled on a TapeStation D5000 Assay (Agilent, D5000 ScreenTape 5067-5588 with D5000 Reagents 5067-5589) and were sequenced on an Element Biosciences AVITI instrument (2x75 Sequencing Kit Cloudbreak Freestyle Medium Output, # 860-00012) in a paired-end

mode (2 x 80 bp). After demultiplexing via bases2fastq, approximately 20 – 30 million read pairs assigned to each sample. All samples passed quality control and were subjected to downstream analysis.

RNA-sequencing and differential gene expression analysis

Alignment and quality control were performed using the NF-Core rnaseq (v.3.18.0) pipeline using the star-salomon aligner (1). Alignment was performed against the human genome assembly GRCH38 and Ensembl gene annotation v114. Unique molecular identifiers were used according to the library manufacturer's instructions. Differential gene expression analysis was performed using the NF-Core differential (v.1.5.0) pipeline (2). Enrichment was performed for up- and down-regulated genes separately using over-representation analysis implemented in the gseapy (v1.1.8) Python package (3). Differential gene expression results were filtered for the enrichment analysis using the thresholds: adjusted p-value ≤ 0.05 and abs (log2FoldChange) > 1. The Reactome Database (v2024), as provided by enrichR (https://maayanlab.cloud/Enrichr/), was used.

Infection serology and autoantibodies analyses

Serum analysis for antibodies against the vaccination status was performed at the Institute of Medical Microbiology, Virology and Hygiene, University Medical Center Hamburg-Eppendorf. Autoantibodies analysis was performed at the Institute of Immunology, University Medical Center Hamburg-Eppendorf.

Plasma cytokine analyses

Serum cytokines were measured using a LUMINEX multianalyte assay kit (Human XL Cytokine Fixed Panel; #LKTM014B, R&D Systems). The protocol was performed according to the manufacturer’s instructions in a 96-well plate with 25 µl of supernatant for cellular protein quantification. Protein quantification was done using the Bio-Plex-System 200 (Bio-Rad Laboratories GmbH) and the Bio-Plex Manager™ 4.1.1 software (Bio-Rad Laboratories GmbH), measured in mean fluorescence intensity (MFI). The MFI values were normalized using protein lysate concentrations measured according to the Bradford method. Serum IFNα and IFNβ levels were measured using VeriKine-HS Human IFN-α All Subtype ELISA Kit (#41115, PBL Assay Science) and VeriKine-HS™ Human Interferon Beta Serum ELISA Kit (#41415-1, PBL Assay Science), respectively, according to the manufacturer’s instructions.

IFNλ 1/3 (IL-29/IL28B) was measured using Human IL-29/IL-28B (IFN-lambda 1/3) DuoSet ELISA (#DY1598B-05, R&D Systems). Cell supernatant IL-6 protein was measured using Human IL-6 DuoSet ELISA (#DY206-05, R&D Systems) and TNF protein was measured using Human TNF-alpha DuoSet ELISA (#DY210-05, R&D Systems). Absorbance was read at 450 nm with the Safire2™ microplate reader (TecanTechnologies). Supernatant of stimulated B cells was collected on day 0, 2, 4 and 7. IgG and IgM production was measured using the ELISA Flex Human IgG (#3850-1AD-6, Mabtech) and IgM (#3880-1AD-6, Mabtech) kit according to the manufacturer's instruction. Samples were measured with the PlateDirect A96 Plate Reader (Mettler Toledo) at 405 nm. A standard curve was run for each plate and the samples' concentrations were interpolated from them.

RNA isolation for quantitative Real-Time PCR analyses

Trizol-resuspended PBMCs were thawed on ice and chloroform was added to extract the RNA from the cells. After a centrifugation step, the aqua phase was loaded on the columns of RNeasy Mini Kit (#74104, Qiagen), following the manufacturer's instructions for RNA precipitation. Synthesis of cDNA was performed using the qScriptTM cDNA Synthesis Kit kit (#95047-100, QuantaBiosciences) and qPCR was performed using the Quantifast SYBR Green supermix (#204154, Qiagen) in a LightCycler® 96 System (Roche), according to the manufacturer's instructions. *HERC5, IFI6, IFI44, IFI44L, IFIT1, IFIT3, IFITM3, IL6, IRF5, IRF7, ISG15, MX1, MX2, RELA, OAS1, OAS2, OAS3, PLSCR1, STAT1, TLR8, TNFA* expression levels were measured by qPCR. The threshold cycle (Ct) was calculated using the mean of two technical duplicates and normalized to a stable housekeeping mRNA (*GAPDH*). All primer sequences used in the study are listed in Supplemental Table 3.

Generation of BlaER1 *TLR8*$^{-/-}$ monocytes expressing the TLR8 protein

BlaER1 *TLR8*$^{-/-}$ monocytes (2.5*106 cells in total) were electroporated with PB_(GOI), pCMV_mcherry_T2A_Flag_hyPBase and PB_rtTA plasmids in a 4:1:1 ratio (6 µg in total) using a Gene Pulser device (BioRad) with the following settings: 265 V, 975 µF, 720 Ω. The rtTA plasmid encodes the reverse tetracycline-controlled transactivator, which binds the doxycycline-responsive promoter and induces expression of TLR8 upon doxycycline treatment. Cell lines expressing TLR8 wild type or

indicated TLR8 point mutants were rested for 2 days after electroporation and selected with blasticidin (10 µg/ml, #A1113903, Thermo Scientific) and puromycin (2.5 µg/ml, #0240.4, Carl Roth).

Generation of TLR8 variant plasmids

Expression plasmids for wild type (WT) *TLR8* gene sequence (NM_138636.5) or A518T, F494L (GOF), G572V (GOF) and D543A (LOF) were generated using a pcDNA3.1(+) backbone plasmid. For the experiments with the HA-tag immunoprecipitation, the HA-tagged oligo was introduced in the C-terminal region of the TLR8 protein after exclusion of the stop codon. All plasmids were purchased from GeneScript Biotech (Netherlands) B.V. Mutations were confirmed by full-plasmid sequencing (Microsynth AG, Switzerland).

Secreted embryonic alkaline phosphatase (SEAP) assay

Transfected HEK BN1 cells were used for the assay. Twenty-four hours post transfection, the cells were stimulated with either 100 ng/mL TL8-506 or 1 µg/mL CL097 for 24 hours. Cell culture supernatants were collected, and SEAP activity was quantified using the QUANTI-Blue™ detection reagent (#rep-qbs2, InvivoGen) following the manufacturer's protocol. Absorbance was measured at 620 nm using a Safire2™ microplate reader (Tecan Technologies). OD values from cells transfected with the empty vector were subtracted from the rest, and the final values normalized to WT.

Cycloheximide chase and proteasomal inhibition assay

Transfected HEK BN1 cells were used for the assay and 48 hours post-transfection, protein synthesis was inhibited using 50 µg/mL cycloheximide – CHX (#C4859, Sigma-Aldrich). TLR8 protein levels were detected upon 8 hours inhibition with or without cycloheximide by western blot.

For the proteosomal inhibition assay, transfected HEK293T cells were used for the assay and 48 hours post-transfection, treated for 6 hours with either cycloheximide (CHX, 50 µg/mL; #C4859, Sigma-Aldrich) to inhibit protein synthesis, MG132 (10 µM, #M7449-200UL, Merck) to block proteasomal degradation, or both. Following the treatment (mock, single, or combined), TLR8 protein levels and ubiquitination were analyzed by western blot in HA-immunoprecipitated lysates. Briefly, cells were washed with PBS on ice, centrifuged for 5 minutes (4 °C, 750 rcf) and lysed using RIPA buffer and

incubation on ice for 10 minutes. Following spin down for 10 minutes (4 ^{o}C, 14000 rcf), 20 µL of anti-HA Magnetic Beads (#88836, ThermoFisher) were added to each sample and incubated on a rotor overnight at 4 ^{o}C. After washing with PBS, pulled down lysates were reconstituted in Laemli buffer and used for western blotting.

Protein analyses by western blot

Cells were cultured as specified, washed with PBS and scraped off in ice-cold cell lysis RIPA-buffer supplemented with complete Mini Protease Inhibitors and PhosStop (Roche). Cell lysates were clarified by centrifugation (18,500 g, 10 minutes, 4 °C) and supernatants were supplemented with sample buffer. Proteins were separated on SDS-polyacrylamide gels and transferred to PVDF membranes using the Transblot Turbo Transfer System (Bio-Rad laboratories). Following blocking (20 mM Tris–HCl, pH 7.4; 150 mM NaCl; 0.1% Tween-20; 5% non-fat dry milk) and washing (20 mM Tris–HCl, pH 7.4; 150 mM NaCl; 0.1% Tween-20), membranes were incubated in primary antibody solution (20 mM Tris–HCl, pH 7.4; 150 mM NaCl; 0.1% Tween-20; 5% BSA or 5% non-fat dry milk) containing the appropriate antibodies: TLR8 (D3Z6J) (#11886S, Cell Signaling, 1:1000 dilution), phospho-NF-κB p65 (Ser536) (#3033S, Cell Signaling, 1:1000 dilution), total-NF-κB p65 (#sc-8008, Santa Cruz, 1:200 dilution), ubiquitin (#19247, abcam, 1:1000 dilution) and HA-Peroxidase (#12013819001, Roche, 1:10000 dilution). γ-tubulin (#ab179503, abcam, 1:2000 dilution) and lactate dehydrogenase (LDHA, #2012, Cell Signaling, 1:1000 dilution) were used as housekeepers. Membranes were washed and incubated with donkey anti-rabbit IgG Horseradish Peroxidase secondary antibody (GE Healthcare; #NA934V; 1:7500 dilution) or with sheep anti-mouse IgG Horseradish Peroxidase secondary antibody (GE Healthcare; #NA931V, 1:7500 dilution). After final washing, proteins were visualized using the ChemiDoc MP Imaging System (Bio-Rad laboratories).

References

1. Patel H, et al. nf-core/rnaseq: nf-core/rnaseq v3.18.0 - Lithium Lynx. 2024. https://doi.org/10.5281/zenodo.14537300.

2. WackerO, et al. nf-core/differentialabundance: v1.4.0 - 2023-11-27. 2023. https://doi.org/10.5281/zenodo.10209675.

3. Fang Z, Liu X, Peltz G. GSEApy: a comprehensive package for performing gene set enrichment analysis in Python. *Bioinformatics*. 2023;39(1):btac757.

Supplemental Figures

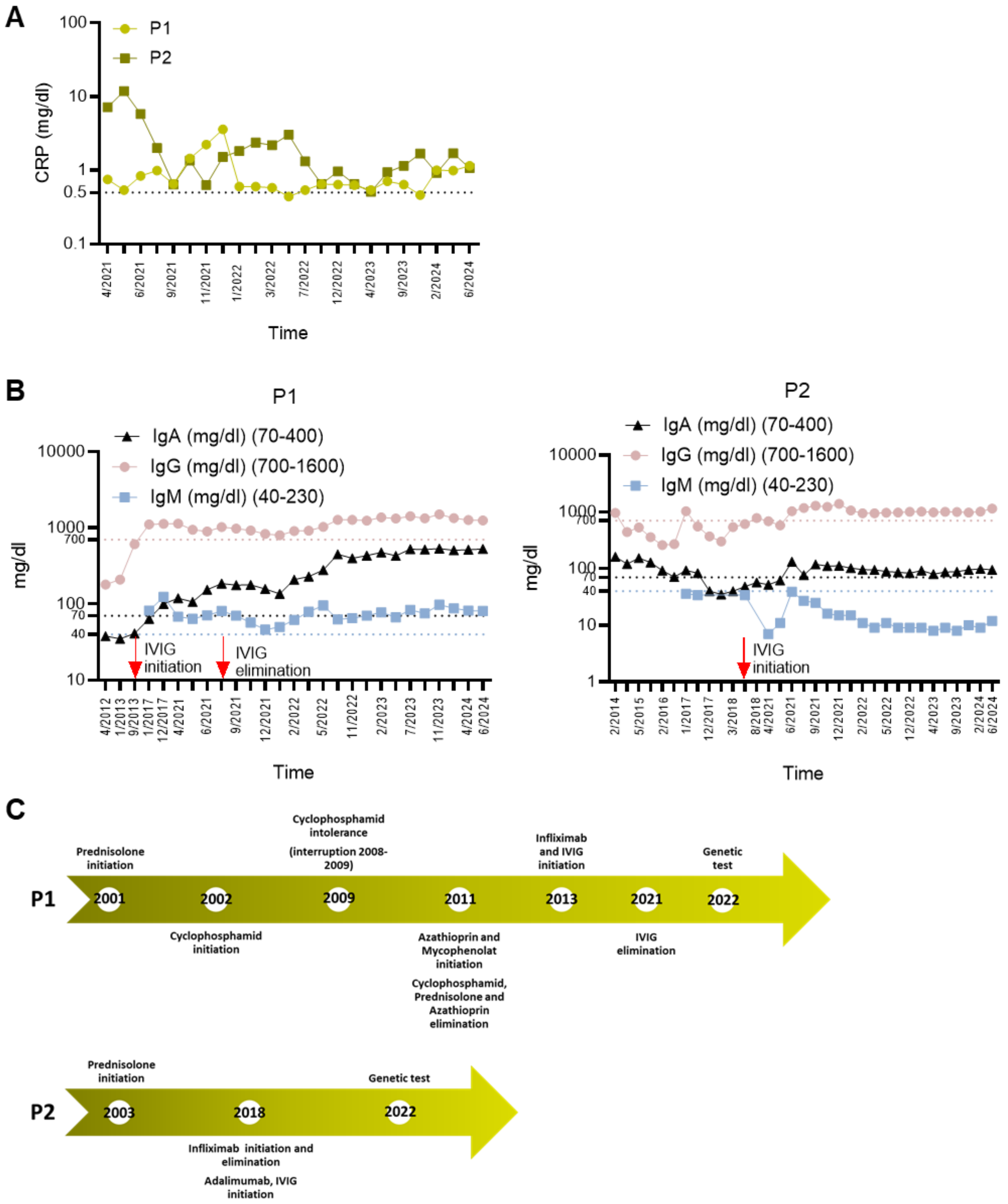

Supplemental Figure 1. Clinical parameter of the affected siblings

(**A**) Time course of CRP measurements for P1 and P2 individuals. (**B**) Time course of IgA/G/M immunoglobulins of P1 and P2 individuals in relation to the IVIG medication. (**C**) Overview of the therapeutic scheme of the P1 and P2 individuals.

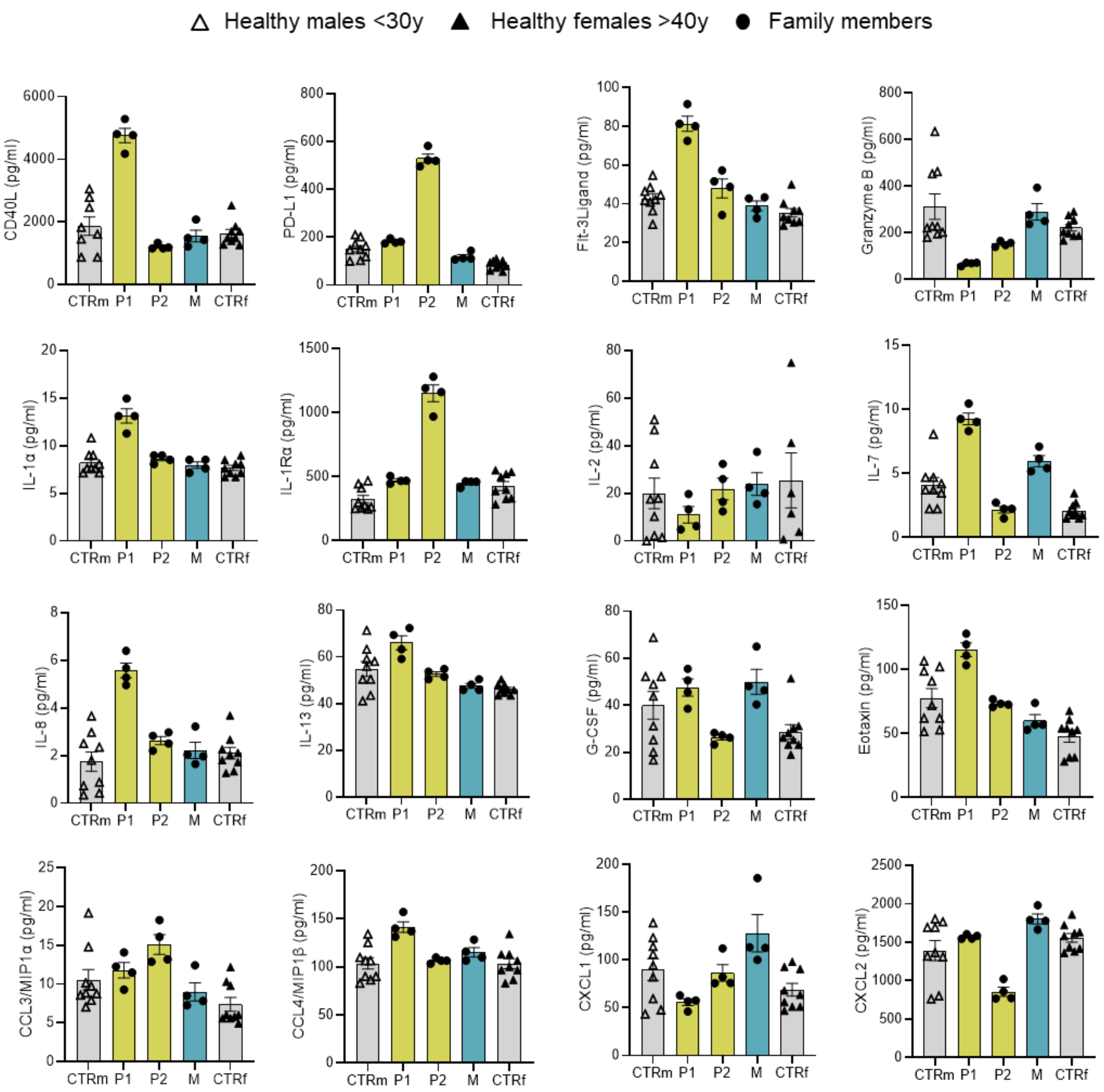

Supplemental Figure 2. Plasma cytokine profile in siblings with the TLR8 A518T variant

Plasma analysis of several pro-inflammatory cytokines, growth factors, and chemokines in family members (P1, P2, and M; performed in quadruplets) compared with healthy male individuals <30 years old (*n* = 3, performed in triplicates) and healthy female individuals >40 years old (*n* = 3-4, performed in triplicates). Data represent mean ± SEM.

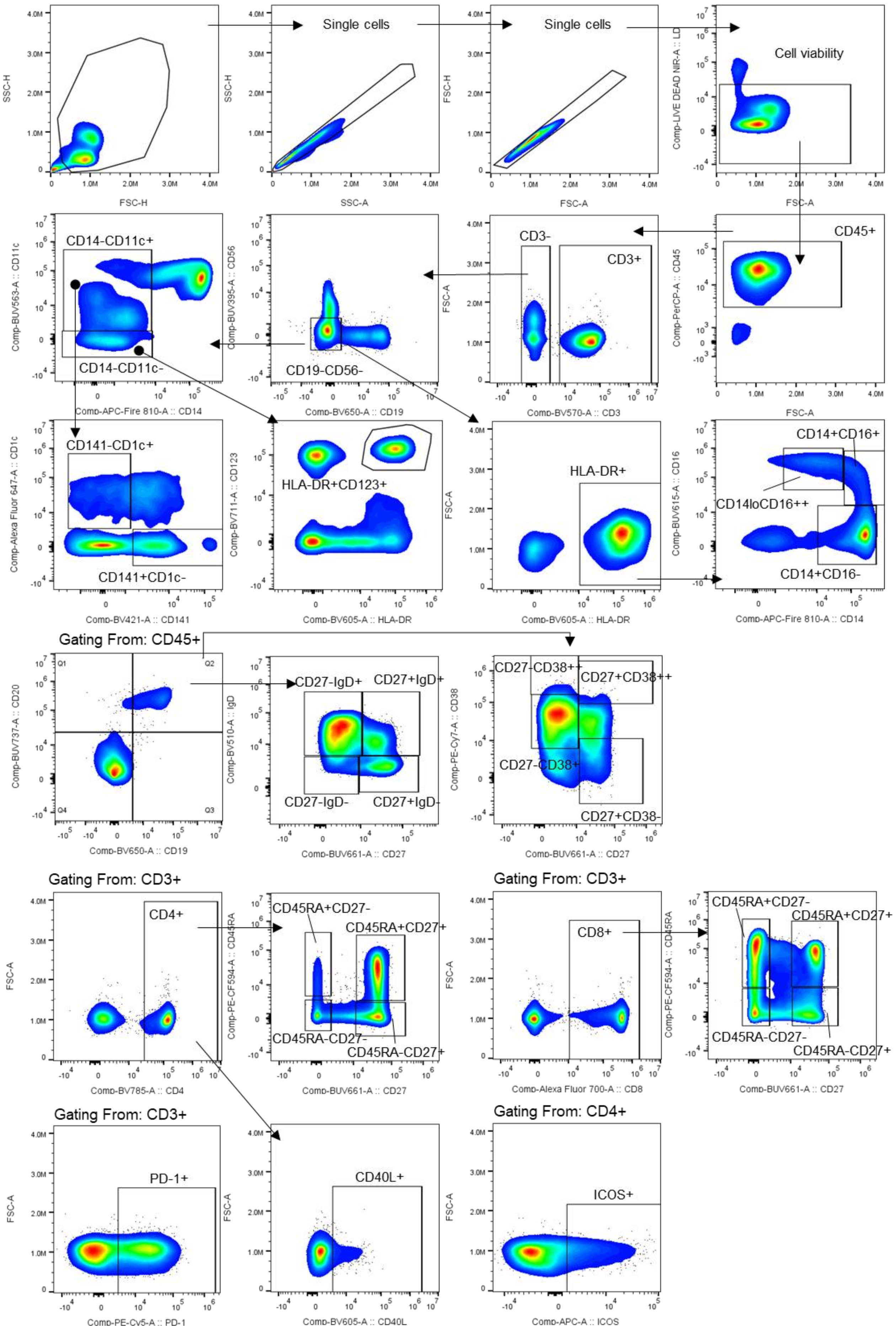

Supplemental Figure 3. Gating strategy for multiparametric flow cytometry analysis.

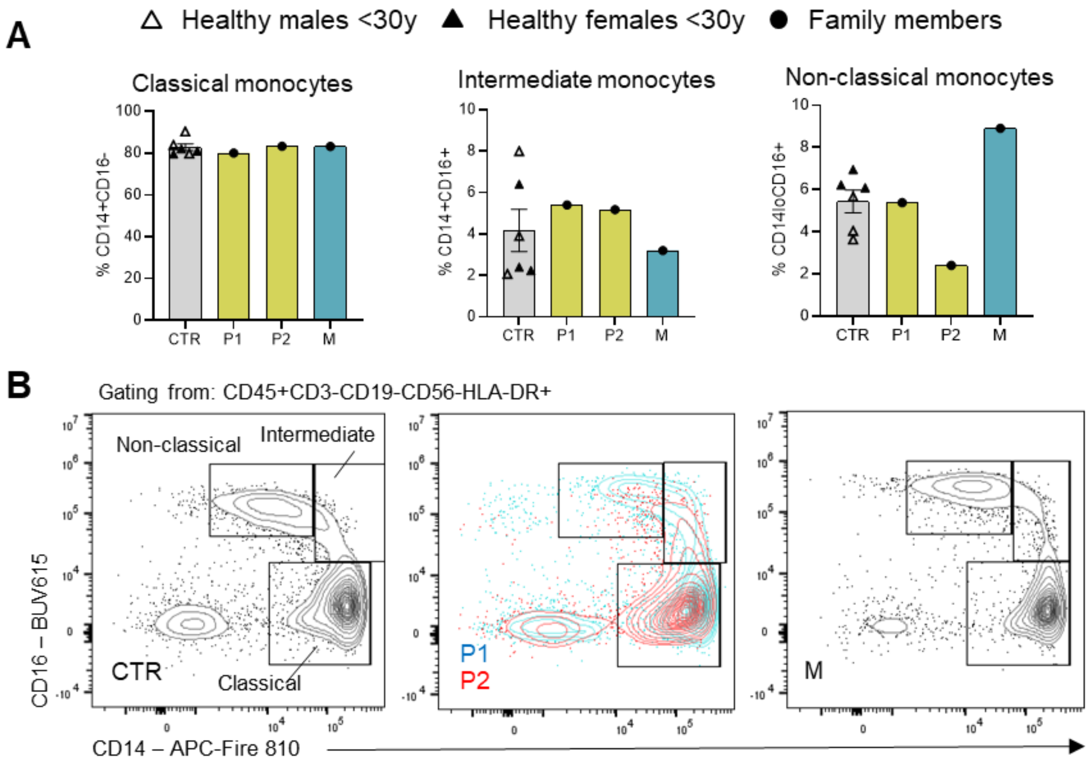

Supplemental Figure 4. Immunological findings in monocytes

(**A**) Percentage of classical, intermediate and non-classical monocytes in family members relative to healthy male (n = 3) and female (n = 3) individuals (CTR). Data represent mean ± SEM for healthy controls. (**B**) Representative flow cytometry plots of monocyte populations in family members relative to healthy male (n = 3) and female (n = 3) individuals (CTR).

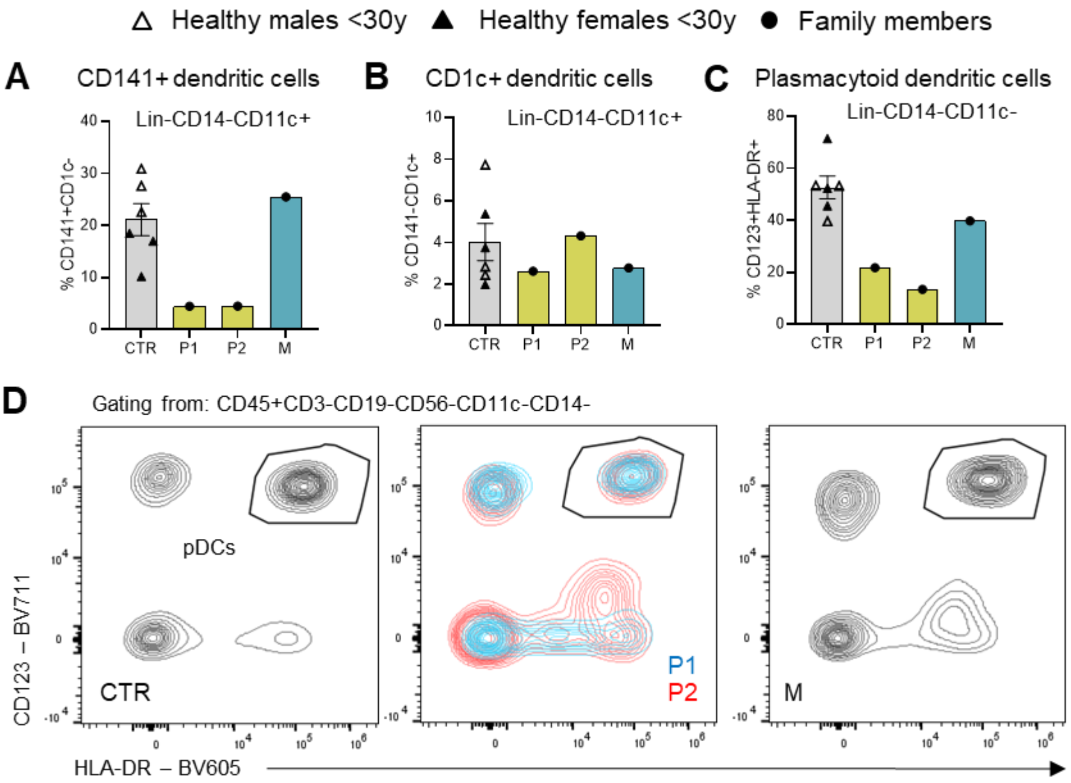

Supplemental Figure 5. Immunological findings in dendritic cells

(**A-B**) Percentages of CD141+ and CD1c+ dendritic cells in family members compared to healthy male (*n* = 3) and female (*n* = 3) individuals (CTR). Data represent mean ± SEM for healthy controls. (**C-D**) Percentage and the representative flow cytometry plots of plasmacytoid dendritic cells in family members relative to healthy male (*n* = 3) and female (*n* = 3) individuals (CTR). Data represent mean ± SEM for healthy controls.

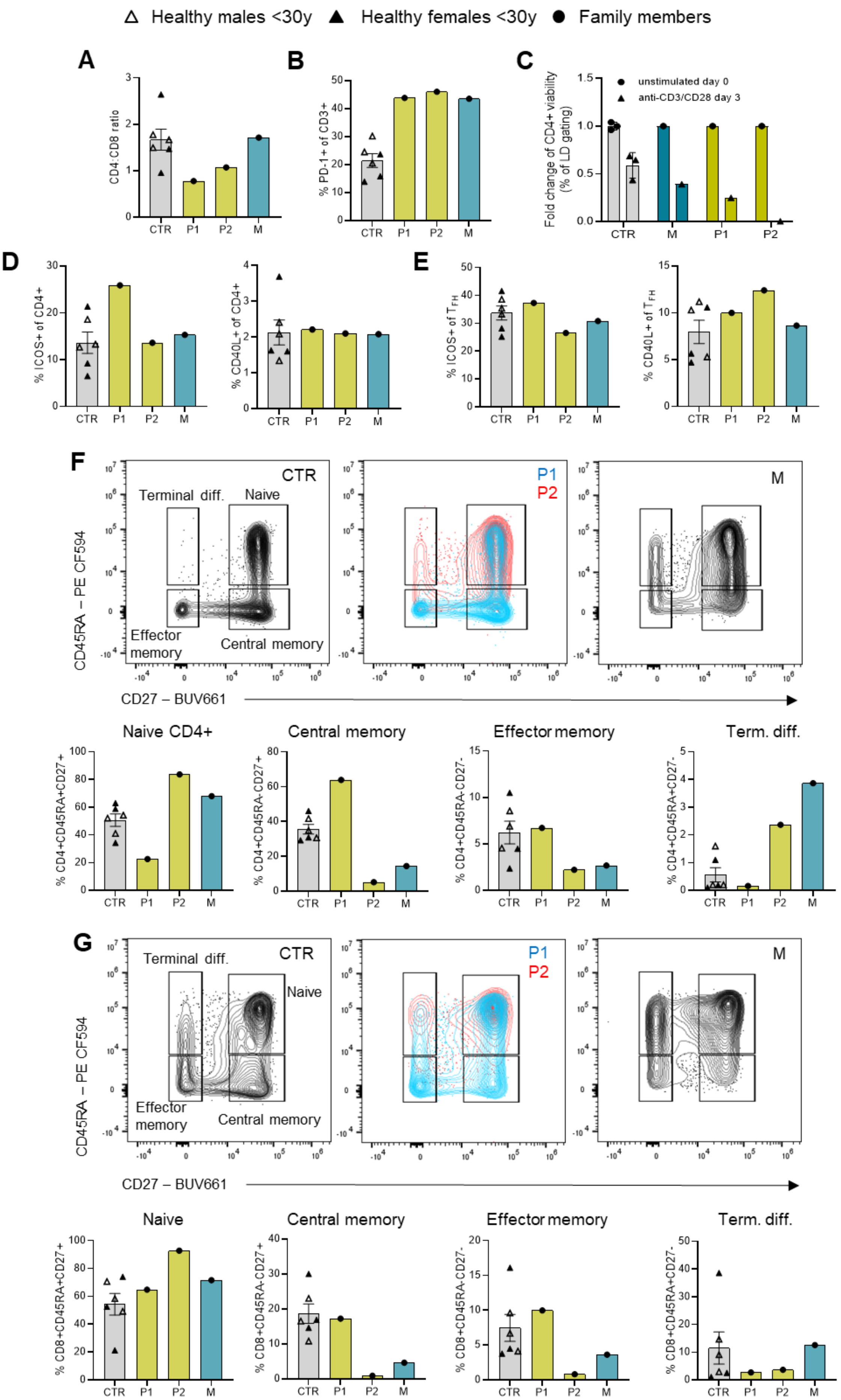

Supplemental Figure 6. Immunological findings in T cells

(**A**) CD4:CD8 ratio in family members relative to healthy male (*n* = 3) and female (*n* = 3) individuals (CTR). (**B**) Percentage of exhausted CD3+PD-1+ T cells in family members relative to healthy male (*n* = 3) and female (*n* = 3) individuals (CTR). (**C**) CD4+ cells viability upon stimulation of isolated T cells with ImmunoCult Human CD3/CD28 T cell activator for 3 days. The data are presented as fold change from the unstimulated day 0 for each donor. (**D-E**) Percentages of the surface markers ICOS and CD40L of PD1+CXCR5+ T_{FH} and CD4+ T cells in family members relative to healthy male (*n* = 3) and female (*n* = 3) individuals (CTR). (**F-G**) Representative flow cytometry plots of CD4 and CD8 populations, along with their frequencies, in family members relative to healthy male (*n* = 3) and female (*n* = 3) individuals (CTR). Data represent mean ± SEM for healthy controls.

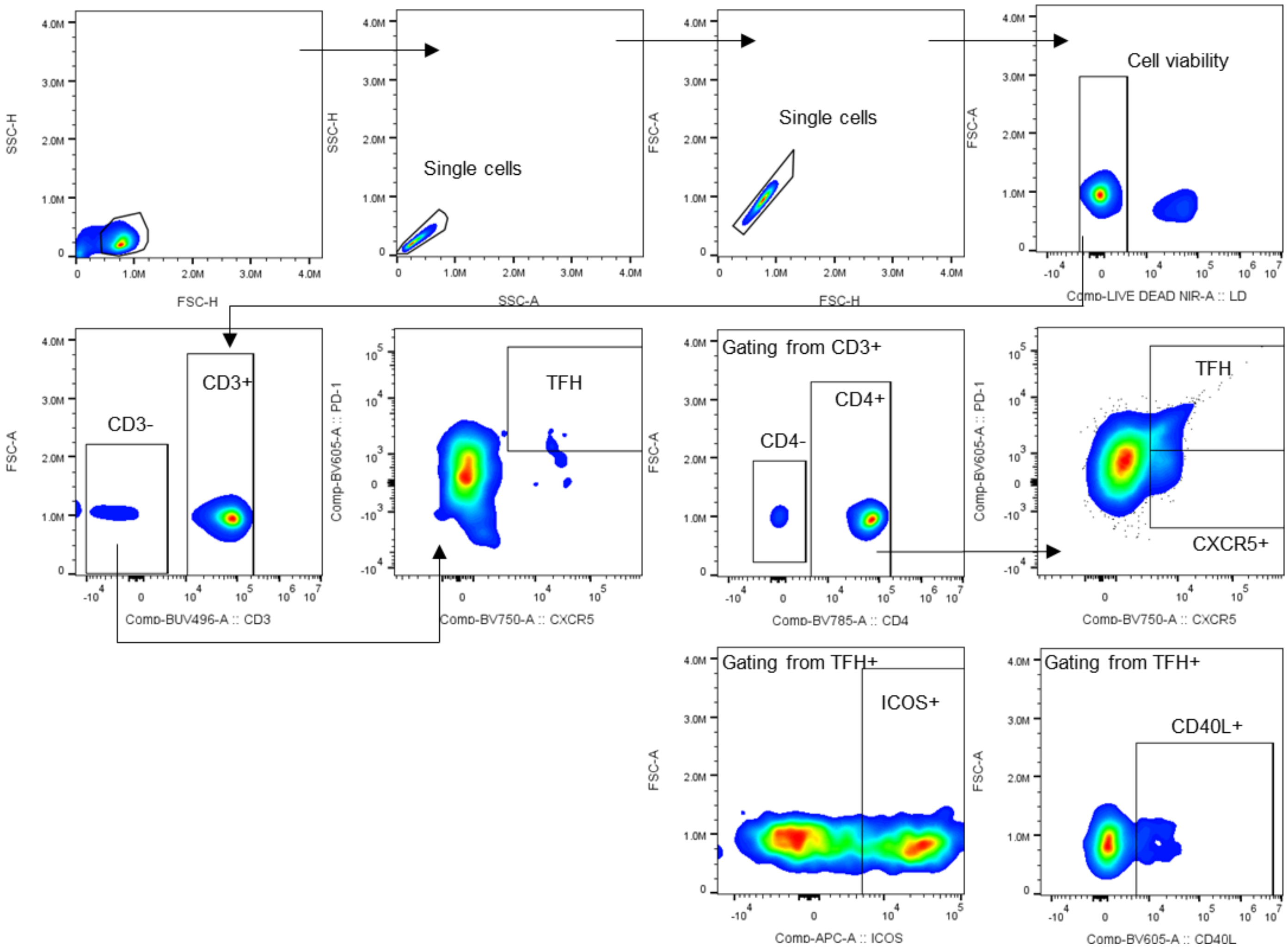

244 **Supplemental Figure 7. Gating strategy of T$_{FH}$ cells**

245 Positively selected CD4+ cells were stained for CXCR5+PD-1+ T$_{FH}$ cells. As a control for gating was
246 used the CXCR5+PD-1- cells in CD3- cells.

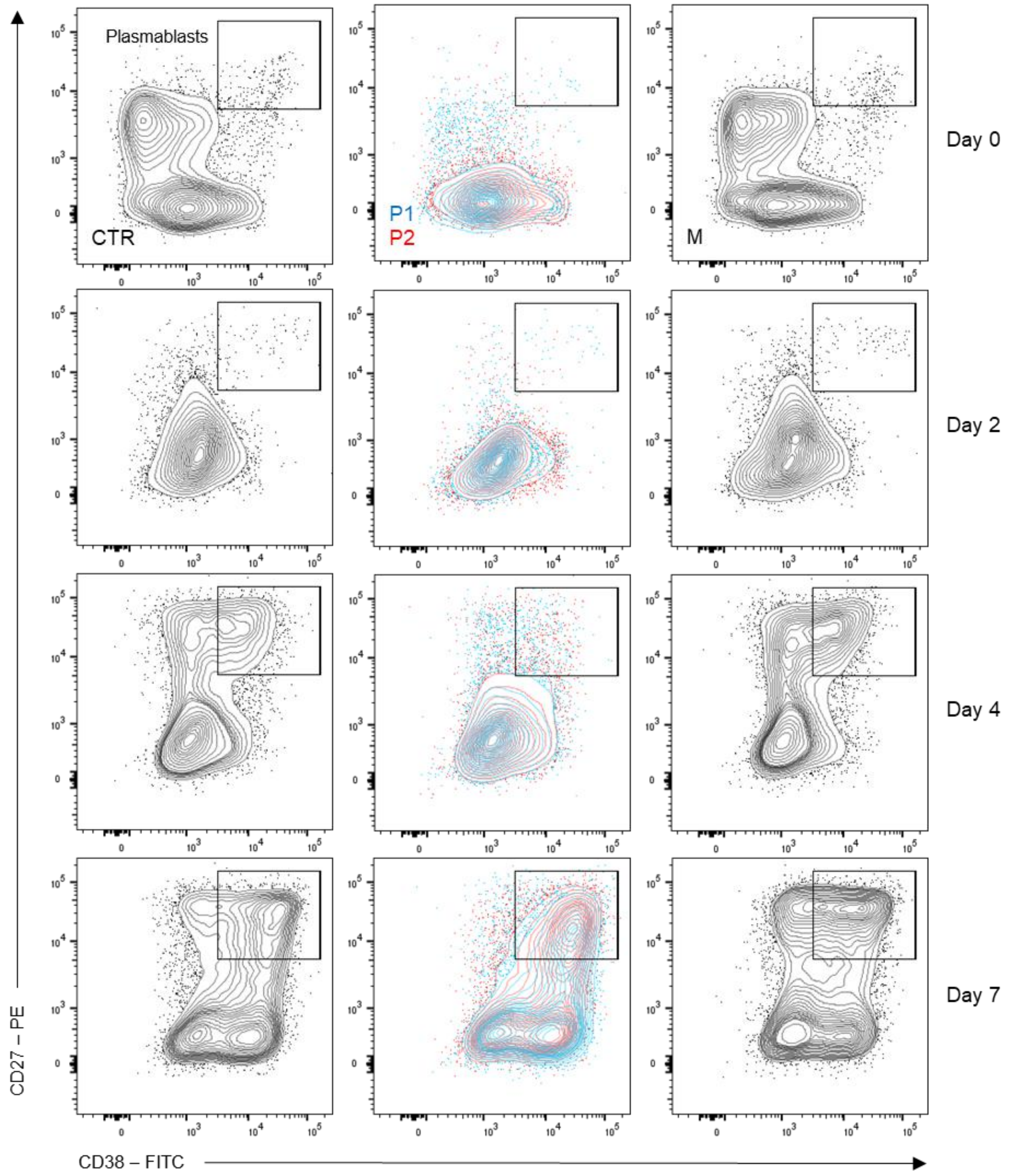

Supplemental Figure 8. Gating for CD27+CD38++ plasmablasts

Representative flow cytometry plots of isolated B cells upon stimulation with CD40L and stained for plasmablasts over time in family members (P1, P2 and M) and healthy male individuals (CTR).

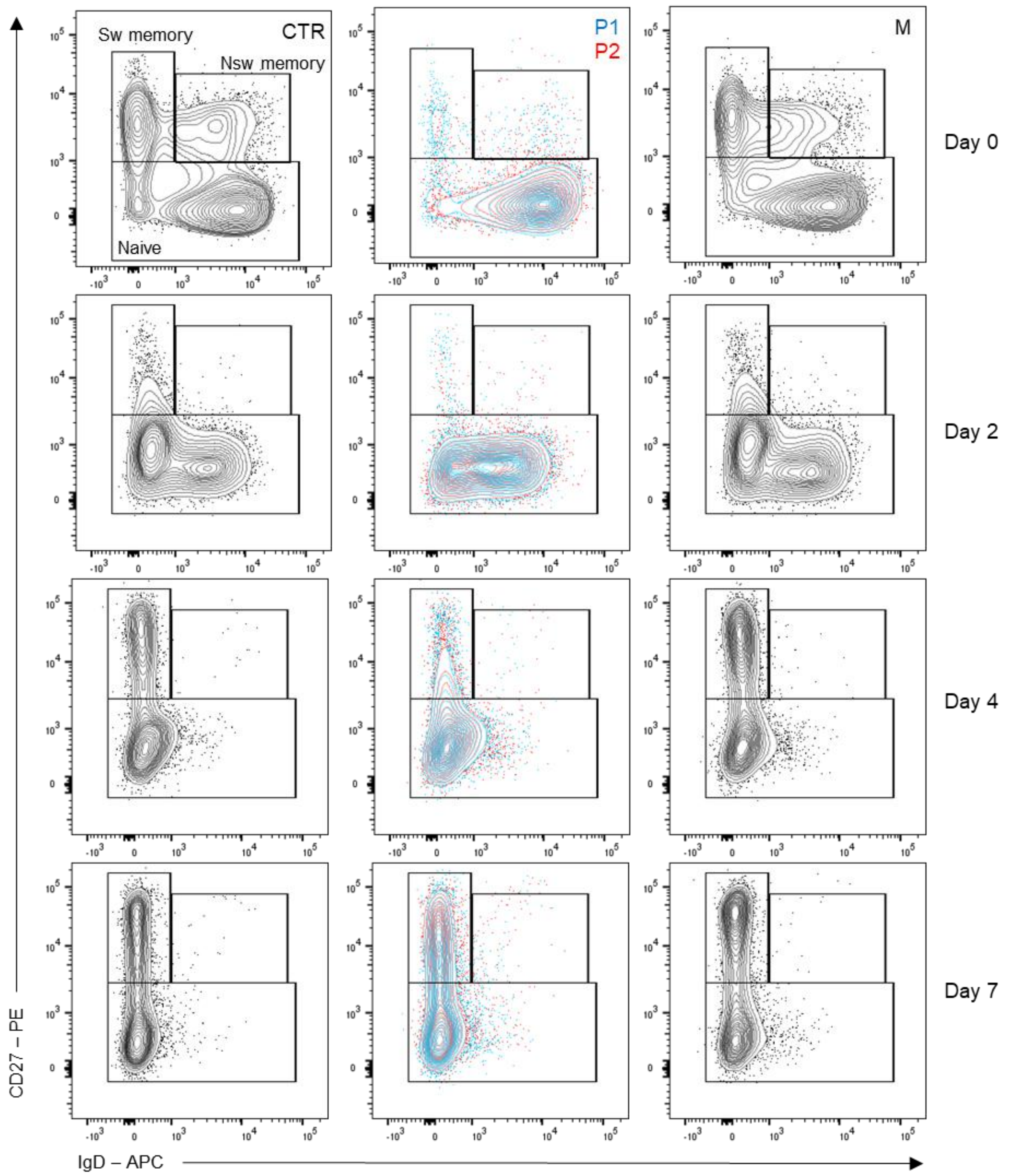

251 **Supplemental Figure 9. Gating for switched memory (sw), non-switched memory (nsw) and naive**
252 **B cells**.

253 Representative flow cytometry plots of isolated B cells upon stimulation with CD40L and stained for
254 sw, nsw memory and naïve B cells over time in family members (P1, P2 and M) and healthy male
255 individuals (CTR).

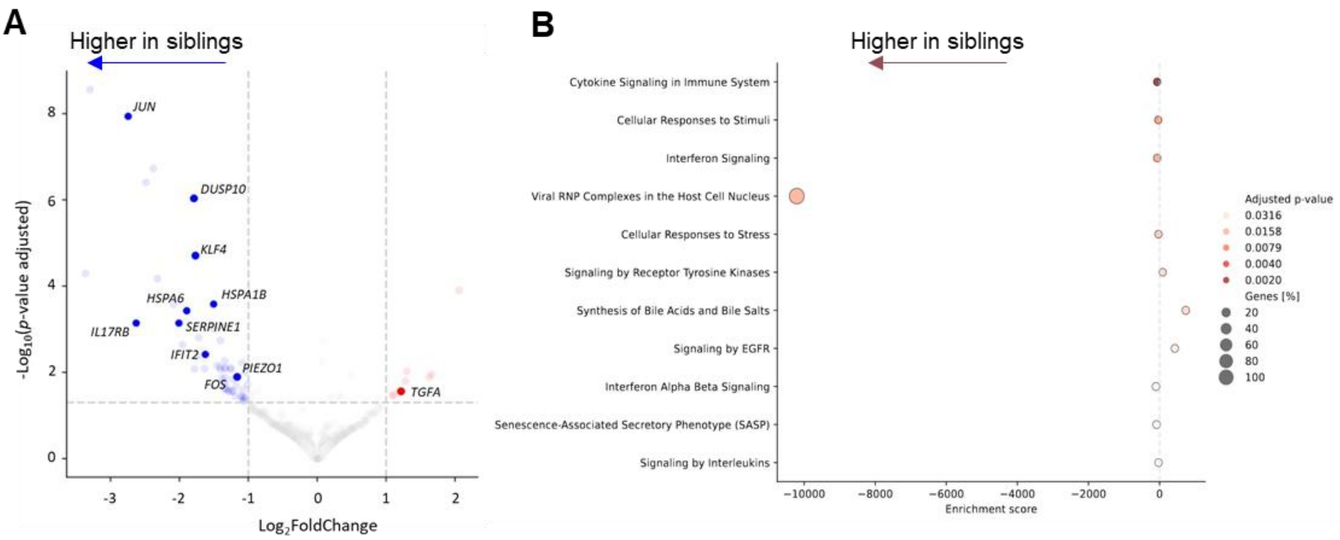

Supplemental Figure 10. Enhanced cytokine and interferon signaling in monocytes from the siblings with the TLR8 A518T variant upon stimulation

(**A**) Volcano plot showing differentially expressed genes in enriched CD14+CD16- monocytes stimulated with 1 µg/mL TL8-506 for 4 hours. Monocytes were isolated from healthy male controls (*n* = 4) and compared to those from the two siblings (P1 and P2). Genes with significant upregulation and downregulation are highlighted based on adjusted *P*-values and fold change thresholds, illustrating transcriptional differences in response to TLR8 stimulation. (**B**) Bubble plot representing the top enriched pathways identified from the differentially expressed genes. Bubble size corresponds to the percentage of genes involved, and color intensity reflects the significance of pathway enrichment.

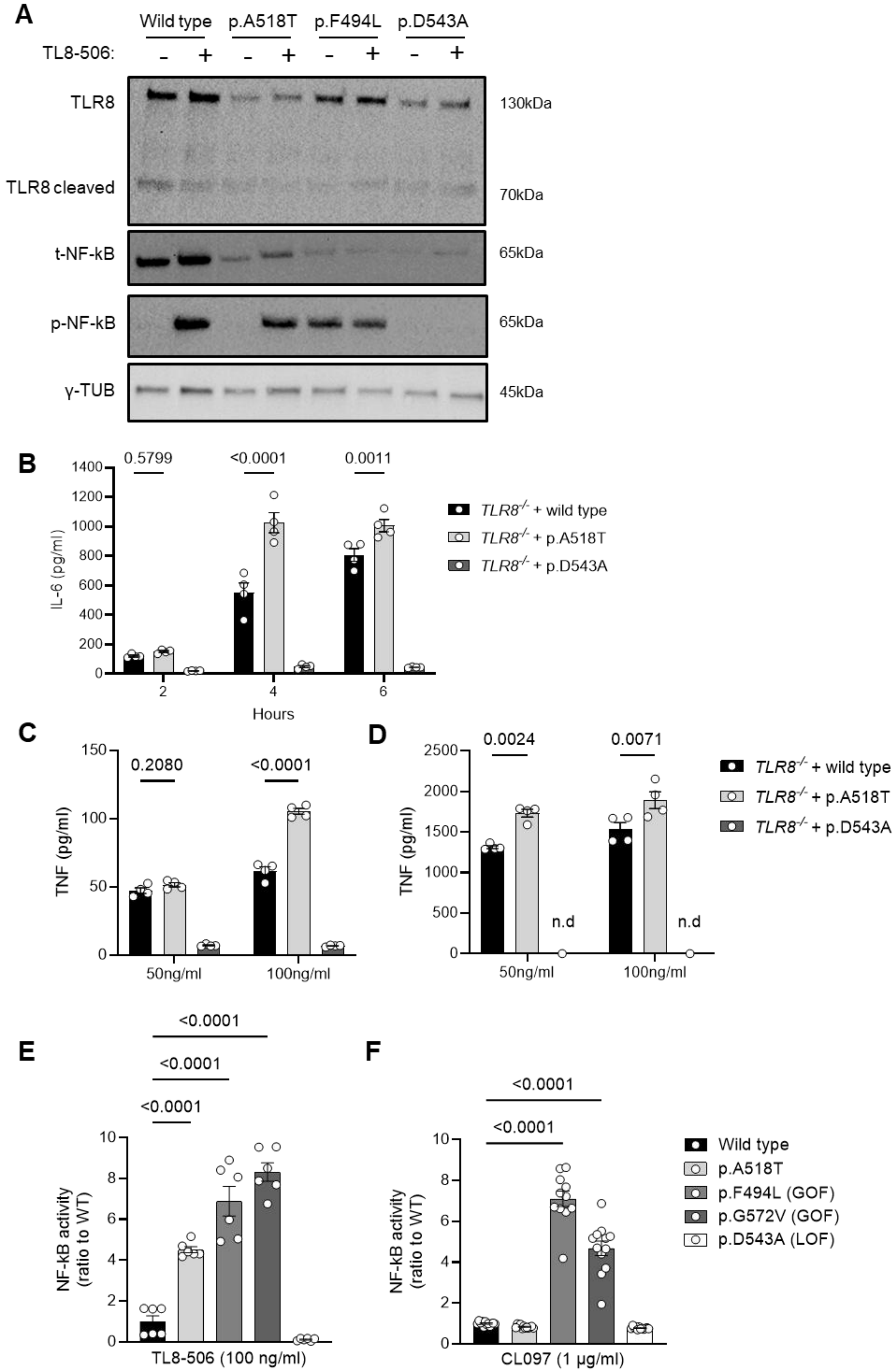
A
Wild type
p.A518T
p.F494L
p.D543A
TL8-506:
- + - + - + - +
TLR8
TLR8 cleaved
t-NF-kB
p-NF-kB
γ-TUB
130kDa
70kDa
65kDa
65kDa
45kDa
B
IL-6 (pg/ml)
0.5799
<0.0001
0.0011
Hours
TLR8-/- + wild type
TLR8-/- + p.A518T
TLR8-/- + p.D543A
C
TNF (pg/ml)
0.2080
<0.0001
50ng/ml
100ng/ml
D
TNF (pg/ml)
0.0024
0.0071
n.d
n.d
50ng/ml
100ng/ml
TLR8-/- + wild type
TLR8-/- + p.A518T
TLR8-/- + p.D543A
E
<0.0001
<0.0001
<0.0001
NF-kB activity (ratio to WT)
TL8-506 (100 ng/ml)
F
<0.0001
<0.0001
NF-kB activity (ratio to WT)
CL097 (1 µg/ml)
Wild type
p.A518T
p.F494L (GOF)
p.G572V (GOF)
p.D543A (LOF)

265

Supplemental Figure 11. Increased NF-κB activation and cytokine production in cells expressing the TLR8 A518T variant upon stimulation

(**A**) Western blot against TLR8, pNF-κB, total NF-κB and γ-TUB in BlaER1 *TLR8*$^{-/-}$ cells with the insertion of the TLR8 wild type protein, or the p.A518T, and the previously published p. F494L (GOF) or p.D543A (LOF) TLR8 variants. Cells are stimulated with 100 ng/ml TL8-506 agonist for 15 minutes. (**B**) IL-6 quantification of BlaER1 *TLR8*$^{-/-}$ cells with the insertion of the TLR8 wild type protein, or the p.A518T and p.D543A (LOF) variants. Cells were stimulated with 100 ng/ml TL8-506 TLR8 agonist at different time points. Data are presented after the subtraction of the unstimulated measurements. Statistical significance between groups was assessed using a two-way ANOVA multiple comparison test. Data represent mean ± SEM of $n = 4$ experiments. (**C-D**) TNF quantification of BlaER1 *TLR8*$^{-/-}$ cells with the insertion of the TLR8 wild type protein, or the p.A518T and p.D543A (LOF) variants. Cells were stimulated with 50 ng/ml or 100 ng/ml TL8-506 agonist for 2 hours (left) and 4 hours (right). Data are presented after the subtraction of the unstimulated measurements. Statistical significance between groups was assessed using a two-way ANOVA multiple comparison test. Data represent mean ± SEM of $n = 4$ experiments (n.d., not detectable values) (**E**) HEK BN1 transfected with wild type TLR8, and TLR8 variants p.A518T, p.F494L (GOF), p.G572V (GOF) and p.D543A (LOF) and stimulated with the TLR8 agonist TL8-506 at 100 ng/ml for 24 hours. NF-κB activity was quantified through the secreted embryonic alkaline phosphatase (SEAP) assay. Statistical significance between groups was assessed using a two-way ANOVA multiple comparison test Data represent mean ± SEM of $n = 3$ experiments run in duplicates. (**F**) HEK BN1 transfected with wild type TLR8, and TLR8 variants p.A518T, p.F494L (GOF), p.G572V (GOF) and p.D543A (LOF) and stimulated with the TLR7 agonist CL097 at 1µg/ml for 24 hours. NF-κB activity was quantified through the secreted embryonic alkaline phosphatase (SEAP) assay. Statistical significance between groups was assessed using a two-way ANOVA multiple comparison test Data represent mean ± SEM of $n = 3$ experiments run in quadruplicates. Differences between groups were considered significant at *P* values less than 0.05.

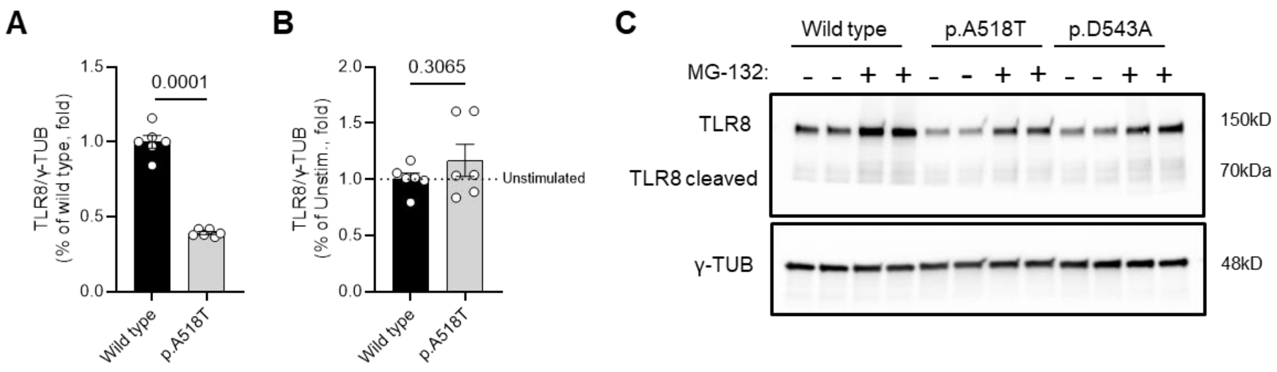

Supplemental Figure 12. Reduced basal and stimulated protein levels of the TLR8 A518T variant

(**A**) Ratio of TLR8 to γ-TUB in unstimulated BlaER1 *TLR8*$^{-/-}$ cells with the insertion of the TLR8 wild type protein, or the p.A518T variant. Data are extracted from western blots of Figure 8A. Statistical significance between groups was assessed using Student t-test. Data represent mean ± SEM of $n = 6$ experiments. (**B**) Ratio of TLR8 to γ-TUB in stimulated with 100 ng/ml TL8-506 agonist for 15 minutes of BlaER1 *TLR8*$^{-/-}$ cells with the insertion of the TLR8 wild type protein, or the p.A518T variant. Data are extracted from western blots of Figure 8A. Data are presented as fold change relative to each respective unstimulated condition, which is represented by the dashed line and equals 1. Statistical significance between groups was assessed using Student t-test. Data represent mean ± SEM of $n = 6$ experiments. (**C**) Western blot against TLR8 and γ-TUB in BlaER1 *TLR8*$^{-/-}$ cells with the insertion of the TLR8 wild type, or the p.A518T variant, and the previously published p.D543A (LOF) TLR8 variant. Cells were stimulated with 10 µM MG132 proteasomal inhibitor for 6 hours. Differences between groups were considered significant at *P* values less than 0.05.

Supplemental Tables

Supplemental Table 1. The CADD score

Chrom	X
**Pos**	12920592
**Ref**	G
**Alt**	A
**RawScore**	2.018455
**PHRED**	19.83

Supplemental Table 2. List of flow cytometry antibodies for multiparemetric immunophenotyping

Company	Catalog Number	Fluorochrome	Marker	Isotype	Clone
**BD Biosciences**	612940	**BUV496**	**CD3**	Mouse BALB/c IgG1, κ	UCHT1
	567345	**R718**	**CD8**	Mouse IgG1, κ	HIT8a
	741358	**BUV563**	**CD11c**	Mouse BALB/c IgG1, κ	B-ly6
	751323	**BUV615**	**CD16**	Mouse BALB/c IgG1, κ	B73.1
	612848	**BUV737**	**CD20**	Mouse C57BL/6 IgG2b, κ	2H7
	750167	**BUV661**	**CD27**	Mouse BALB/c IgG1	L128
	560677	**PE-Cy7**	**CD38**	Mouse IgG1, κ	HIT2
	562298	**PE-CF594**	**CD45RA**	Mouse IgG2b, κ	HI100
	744223	**BUV395**	**CD56**	Mouse IgG1, κ	R19-760
	747111	**BV750**	**CXCR5 (CD185)**	Rat IgG2b, κ	RF8B2
**BioLegend**	331510	**Alexa Fluor 647**	**CD1c**	Mouse IgG1, κ	L161
	300436	**BV570**	**CD3**	Mouse IgG1, κ	UCHT1
	300554	**BV785**	**CD4**	Mouse IgG1, κ	RPA-T4
	367156	**APC/Fire 810**	**CD14**	Mouse IgG1, κ	63D3
	363026	**BV650**	**CD19**	Mouse IgG1, κ	SJ25C1
	368506	**PerCP**	**CD45**	Mouse IgG1, κ	HI30
	306030	**BV711**	**CD123**	Mouse IgG1, κ	6H6
	344114	**BV421**	**CD141**	Mouse IgG1, κ	M80
	310826	**BV605**	**CD40L (CD154)**	Mouse IgG1, κ	24-31
	329972	**PE-Cy5**	**PD-1 (CD279)**	Mouse IgG1, κ	EH12.2H7
	367425	**BV605**	**PD-1 (CD279)**	Mouse IgG1, κ	NAT105
	395504	**PE**	**TLR8 (CD288)**	Mouse IgG2a, κ	S16018A
	307640	**BV605**	**HLA-DR**	Mouse IgG2a, κ	L243
	348220	**BV510**	**IgD**	Mouse IgG2a, κ	IA6-2
**Cell signaling**	4886S	**Alexa Fluor 488**	**pNFκBp65 (Ser536)**	Rabbit IgG	93H1
**Invitrogen**	L34976A	**LIVE/DEAD Fixable Near-IR Dead**	**LIVE/DEAD Fixable Near-IR Dead**		

**Miltenyi Biotec**	130-100-719	**APC**	**CD278 (ICOS)**	Recombinant human IgG1	REA192

Supplemental Table 3. List of gene expression primer sequences

**Gene symbol**	**Forward primer**	**Reverse primer**
HERC5	TGCACTCTCAAAAGGTGGTG	CCAGGCCTAGTTGTCCACAT
IFI6	AGGATGAGGAGTAGCCAGCA	TTGGGAGGTTGAGACAGGAG
IFI44	AGCCTGTGAGGTCCAAGCTA	ATCTGCAGCCCATAGCATTC
IFI44L	TATGTGTGTTGGCTGGGAGA	GGGCCTGCATACCTCATAGA
IFITM3	TCGCCTACTCCGTGAAGTCT	CATAGGCCTGGAAGATCAGC
IFIT1	GGCAAAACCAACCGTCTCTA	TTTGAGATGGGGTCTCGTTC
IFIT3	GAACATGCTGACCAAGCAGA	CAGTTGTGTCCACCCTTCCT
ISG15	GAGAGGCAGCGAACTCATCT	CTTCAGCTCTGACACCGACA
MX1	GCCACAAGGCACCTAAGTC	ATTCTCCAAACCTGCGCTCT
MX2	AAGCAGTATCGAGGCAAGGA	TCGTGCTCTGAACAGTTTGG
OAS1	GAGCTCCTGACGGTCTATGC	GTTTCGTGAGCTGCCTTCTC
OAS2	GCTCCTATGGACGGAAAACA	TGAACCCATCAAGGGACTTC
OAS3	GACCTAAGGGATGGCTGTGA	CAGGAAACTGAAGGCTCAGG
PLSCR1	GGTTTACTTTGCAGCGGAAG	ACCAGGAGGAGCTTGGATTT
STAT1	CCGTTTTCATGACCTCCTGT	TGAATATTCCCCGACTGAGC
IRF5	GGGACTGATGTGGAGATGTG	CTCTCCTTCTTGGCCCAAAT
IRF7	TACCATCTACCTGGGCTTCG	AGGGTTCCAGCTTCACAG
IL6	TGCAATAACCACCCCTGACC	TGCGCAGAATGAGATGAGTTG
TNF	CTCTTCTGCCTGCTGCACTTTG	ATGGGCTACAGGCTTGTCACTC
TLR8	CTGTGAGTTATGCGCCGAAGA	TGGTGCTGTACATTGGGGTTG
RELA	TGAACCGAAACTCTGGCAGCTG	CATCAGCTTGCGAAAAGGAGCC
GAPDH	CGGAGTCAACGGATTTGG	TGATGACAAGCTTCCCGTTC
