# Supplementary material for: Structural modeling and functional characterization of a novel gain-of-function TLR8 variant causing severe inflammatory syndrome
Source: JCI Insight. 2026 Feb 23;11(4):e187422. doi: 10.1172/jci.insight.187422 (PMC12956005; doi:10.1172/jci.insight.187422)

Full unedited gel for Figure 8A

TLR8
Cleaved TLR8

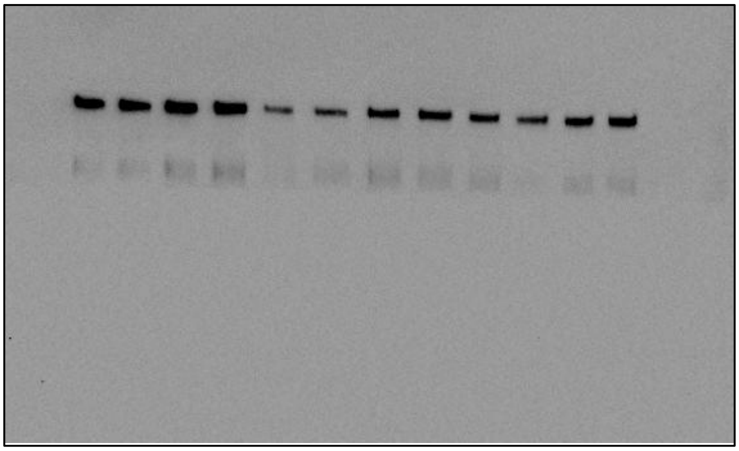

p-NFκB

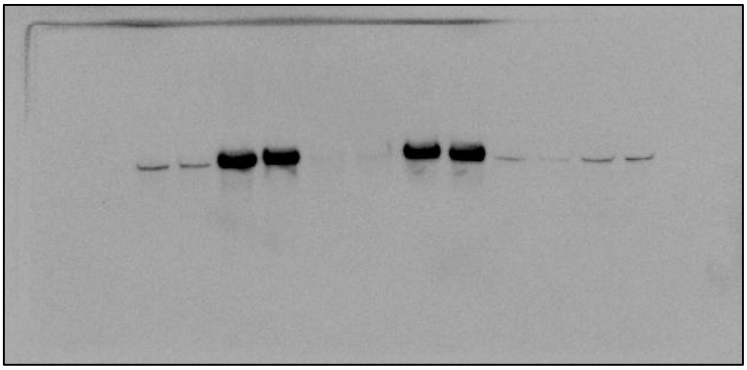

t-NF-κB

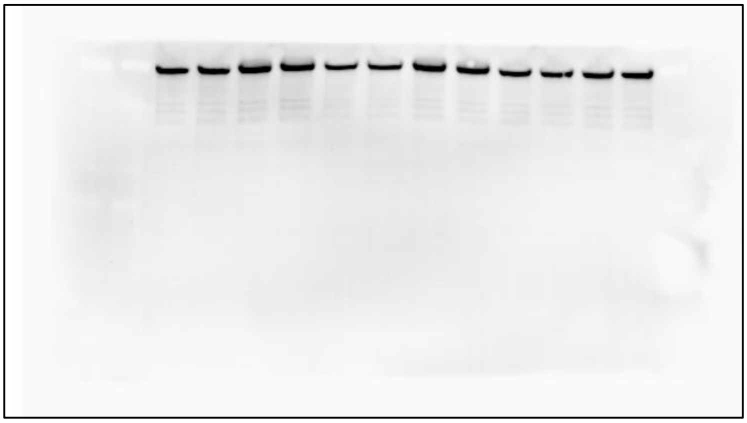

γ-TUB

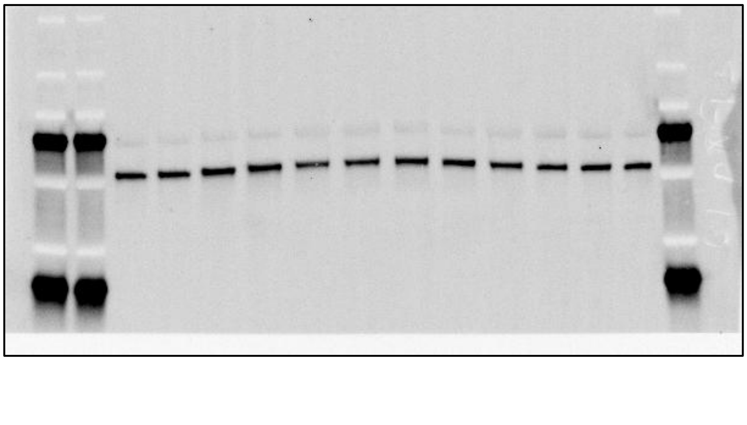

Full unedited gel for Figure 9A

TLR8

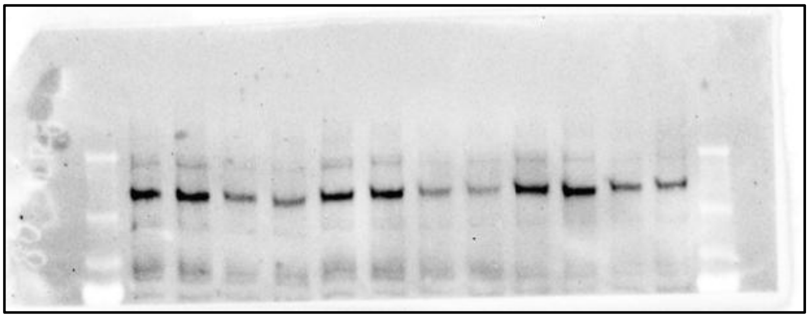

γ-TUB

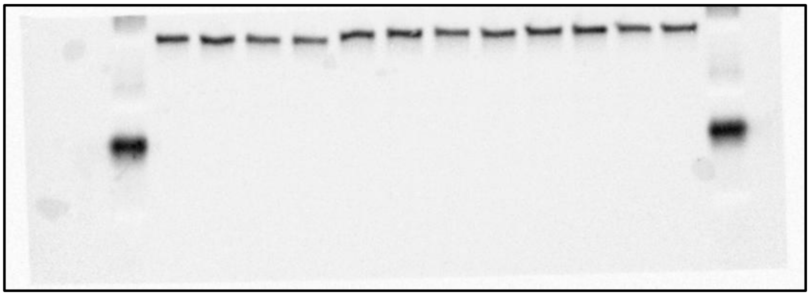

LDHA

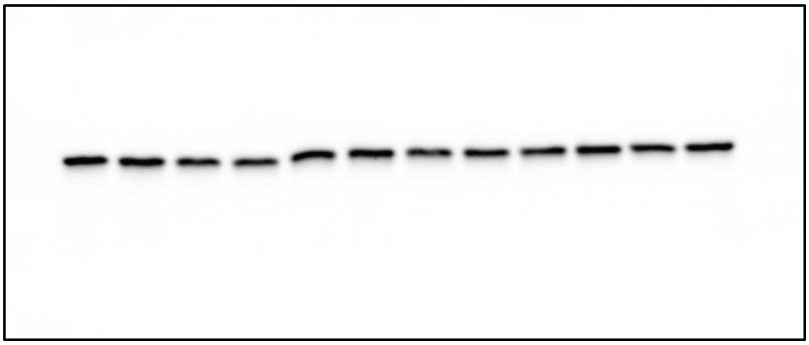

Full unedited gel for Figure 9B

Ubiquitin

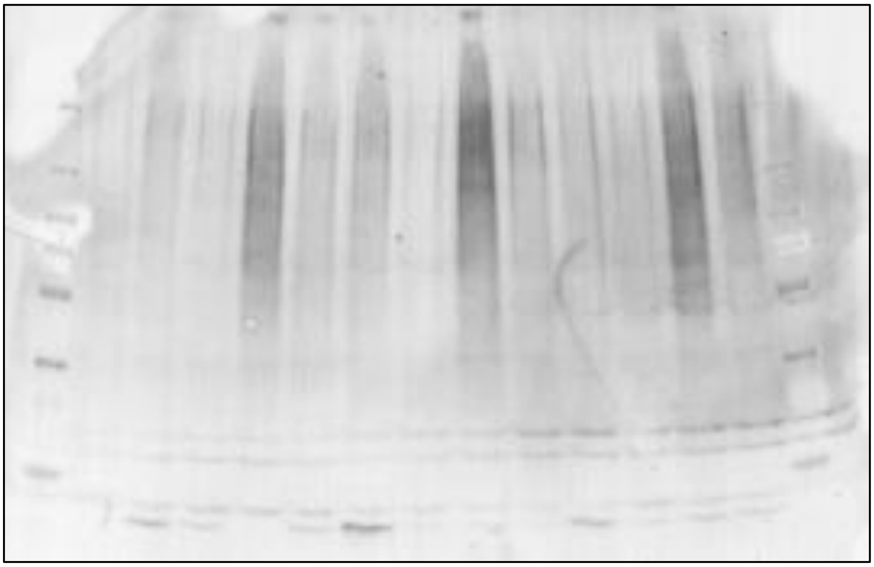

TLR8

Cleaved TLR8

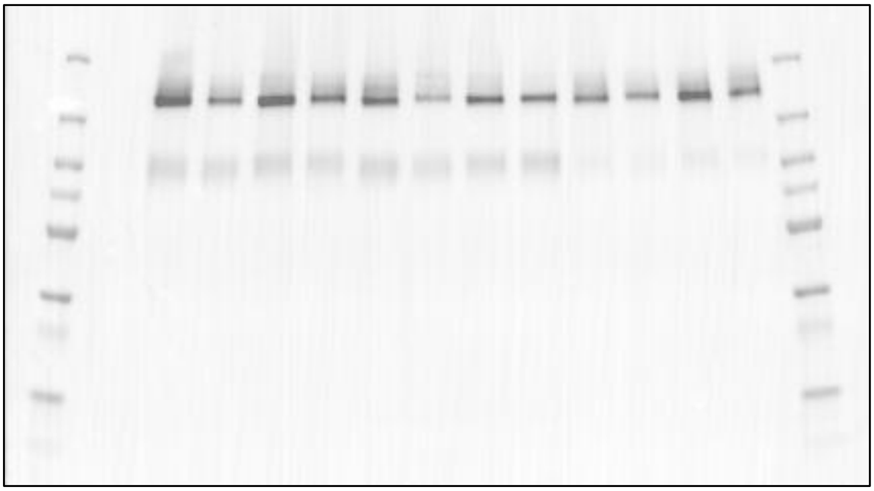

HA-tag

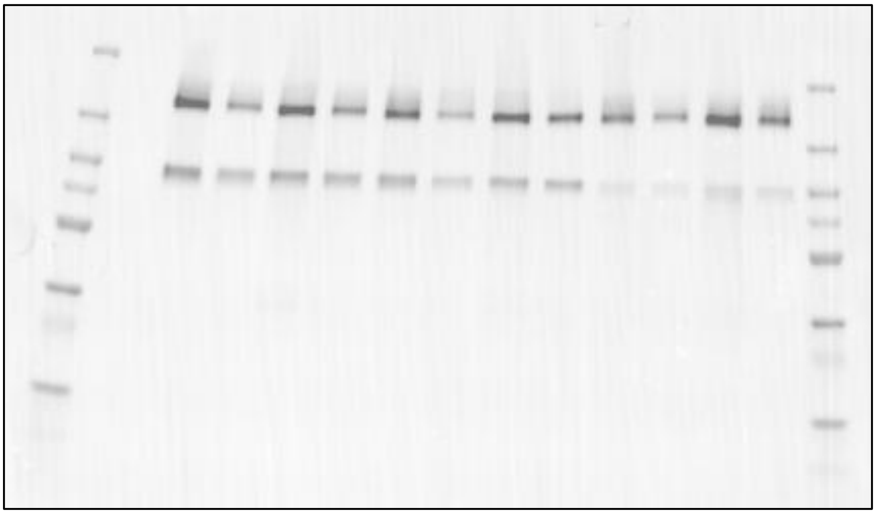

Full unedited gel for Supplemental Figure 11A

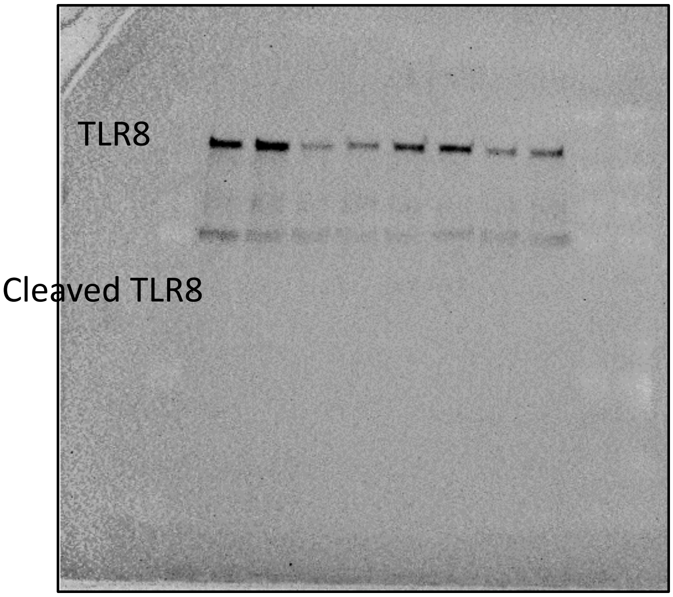

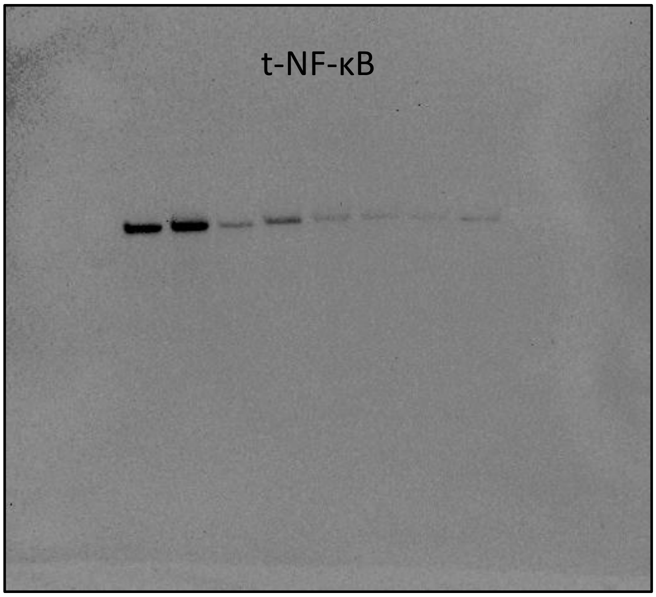

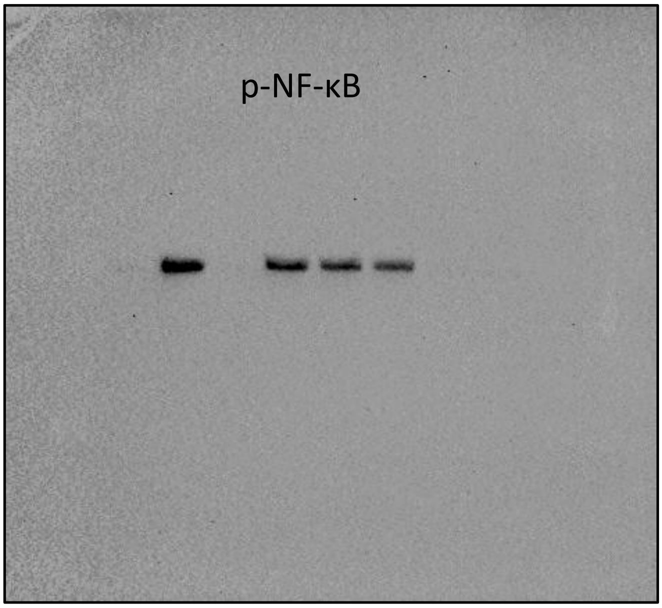

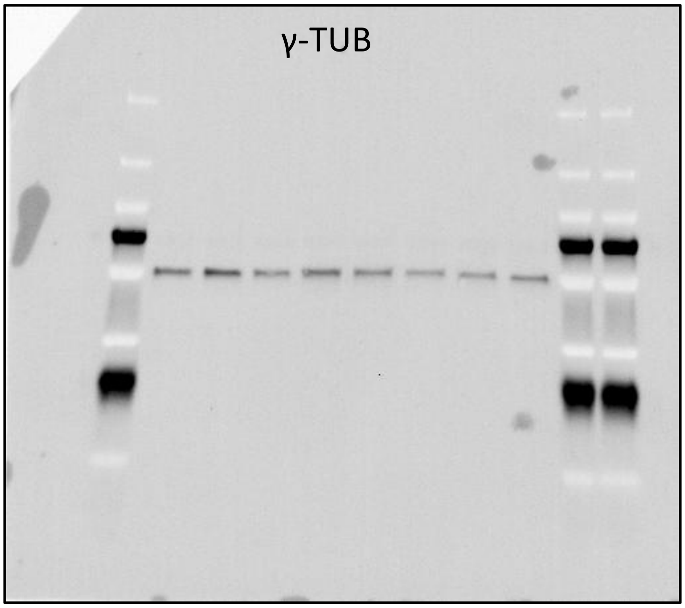

Full unedited gel for Supplemental Figure 12C

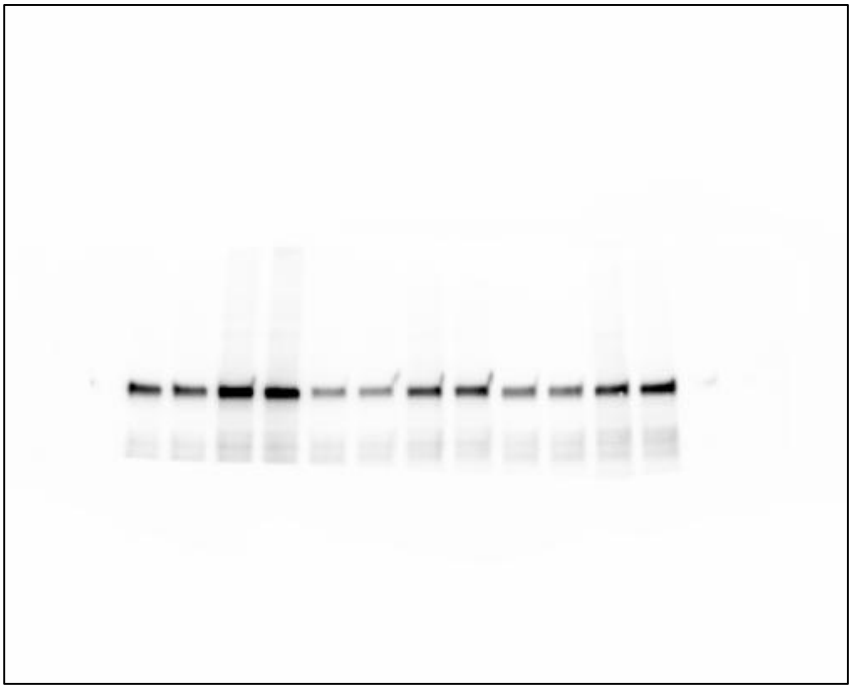

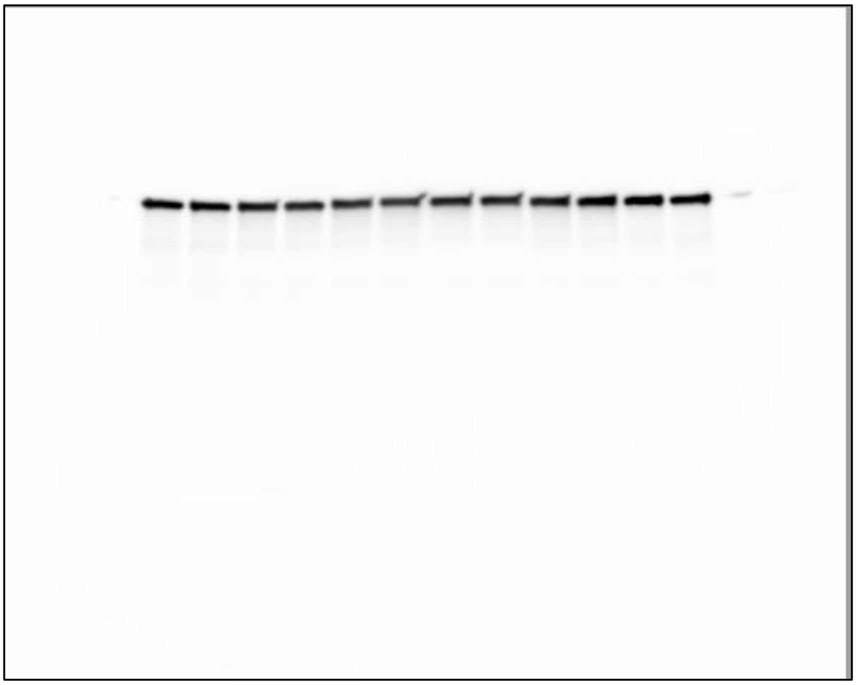

Supplement: Unedited blot and gel images [file jciinsight-11-187422-s277.pdf]
